# Green infrastructure has weak conceptual links with efficient biodiversity conservation

**DOI:** 10.1007/s13280-025-02149-1

**Published:** 2025-02-22

**Authors:** Johan Ekroos, Maria von Post, Anna S. Persson, Martin Stjernman, Ola Olsson

**Affiliations:** 1 Department of Biology, Lund University, Ecology Building, Kontaktvägen 10, 223 62 Lund, Sweden; 2 Centre for Environmental and Climate Science, Lund University, Ecology Building, Kontaktvägen 10, 223 62 Lund, Sweden; 3 Department of Agricultural Sciences, University of Helsinki, Yliopistonkatu 3, P.O. Box 4, 00014 Helsinki, Finland; 4 Helsinki Institute of Sustainability Science, HELSUS, University of Helsinki, Helsinki, Finland

**Keywords:** Biodiversity policy, Conservation prioritisation, Ecological networks, EU Nature Restoration Law, Evidence-based conservation

## Abstract

To reverse biodiversity loss, creating resilient ecological networks has been promoted in policy and practice. However, we argue that emphasising networks within policy without clear conservation prioritisations may direct focus away from ecological processes important for population persistence. We studied the rationale for resilient ecological networks represented in a policy context, by reviewing research related to biodiversity within the concept of green infrastructure in European policy. We compared this outcome with underlying empirical evidence for effects of landscape properties on ecological processes relevant to population persistence. We show that interventions within green infrastructure research partly diverge from evidence of efficient conservation derived from empirical studies, likely linked to an insufficient acknowledgement of ecological processes determining long-term conservation of populations in GI policy and research. We discuss possible implications for biodiversity conservation and argue for upcoming policies to better integrate scientific evidence and underlying ecological processes to improve biodiversity outcomes.

**Supplementary Information:**

The online version contains supplementary material available at 10.1007/s13280-025-02149-1.

## Introduction

Biodiversity loss continues despite efforts to reverse the negative trend (IPBES 2019). Developing and strengthening resilient ecological networks has been promoted as an efficient solution for long-term conservation of species and ecosystems, particularly in the context of ongoing climate change (Lawton et al. 2010; European Commission 2011, 2013; Oliver et al. 2015; Albert et al. 2017; Isaac et al. 2018). Well-functioning ecological networks can enhance population persistence and recolonisation or recovery after local extinctions driven by, e.g. extreme weather events or other stochastic or projected effects. They could also facilitate range shifts of populations by supporting movement between the nodes in the network (Hodgson et al. 2016; Albert et al. 2017; Isaac et al. 2018).

**Fig. 1:**
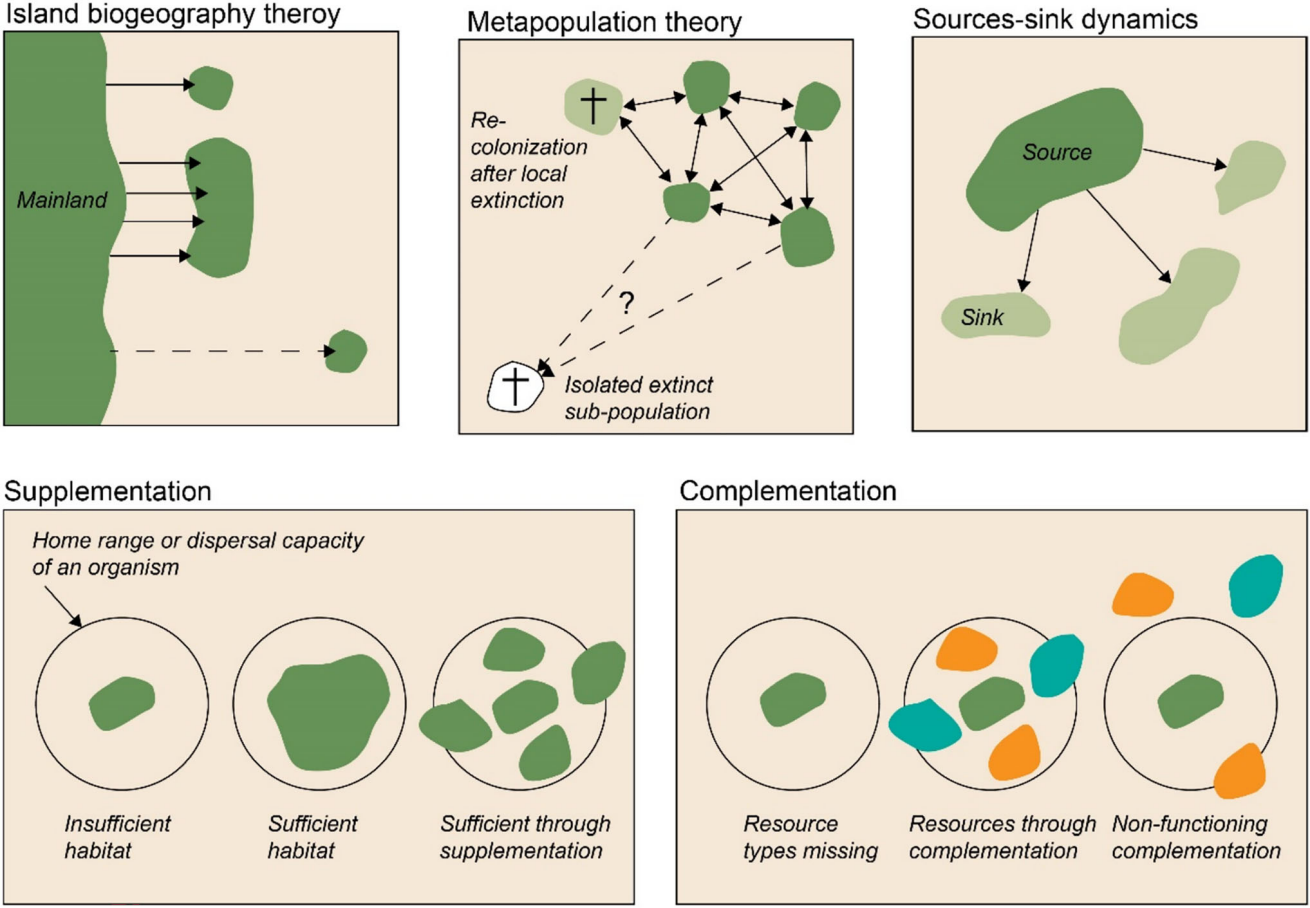
Theoretical frameworks of relevance for spatial planning and prioritisation of efficient conservation interventions for the creation of resilient ecological networks. MacArthur and Wilson (1967) presented the island biogeography theory (**a**) describing how the state of equilibrium between number of colonisations and extinctions of species depends on the size of the island (patch size) and the distance from the mainland (degree of isolation), with equilibrium shifts leading to more species on bigger islands closer to the mainland. The theory has been highly influential for the scientific development of the field of conservation biology. However, it was recognised early on that it does not account for interspecific variation and effects of between-habitat interactions, caused by variation in landscape configuration and composition (see for instance Simberloff 1974; Zimmerman and Bierregaard 1986; Lomolino 2000). Influential theories attempting to capture such effects include, e.g. the metapopulation theory (Levins 1969; Hanski 1999) (**b**), and the theory of source and sink dynamics (Pulliam 1988; Dias 1996) (**c**). The importance of metapopulation theory and source-sink theory increases in contexts involving highly fragmented, mosaic landscapes (Pulliam 1988; Hanski 1999 ), where sufficient habitat and available resources across entire landscapes may become critical. In a metapopulation context, long-term population viability and persistence is determined by the balance between colonisation, extinction and recolonisation of local habitats in a habitat patch network, where any one of the sub-populations could go extinct at any time due to stochastic effects (Hanski 1999). Source-sink dynamics in turn explains how differences in habitat quality results in demographic effects, where populations in high quality habitats (sources) produce a surplus of individuals, while populations in poor quality habitats (sinks) suffer from reproduction deficiency but through immigration from the sources still can persist (Pulliam 1988; Dias 1992). These two ecological mechanisms tend to emphasise between-year dynamics and ecological processes, most notably dispersal and survival in the case of meta-populations and additionally reproductive success in the case of source-sink systems (Ekroos et al. 2016). Another influential theory is the theory of landscape complementation and supplementation, that in contrast considers how resources needed for population persistence are distributed within patchy or fragmented landscapes, such that individuals need to move between patches to acquire essential resources of the same kind by supplementation (**d**), or to acquire enough resources of different kind by complementation (**e**) (Dunning 1992). Under this framing, the ecological timescale emphasises mechanisms that play out within seasons, linked to resources needed for survival and reproduction (Ekroos et al. 2016). Illustration: Anna S. Persson

The creation of resilient ecological networks to protect biodiversity has been suggested in various contexts, such as spatial planning (Benedict and McMahon 2006), practical conservation management (Lawton et al. 2010), and in recent EU policy (European Commission 2013), including the EU Nature Restoration Law ((EU) 2024/1991). In the EU, the European Strategy on green infrastructure (hereafter GI; European Commission 2013) draws in part on the logic of resilient ecological networks to enhance biodiversity conservation through the promotion of ecological networks to benefit people and nature (von Post et al. 2023). Ideally, defining resilient ecological networks in spatial planning and prioritisation of conservation interventions should be based on an explicit consideration of their influence on ecological processes that determine long-term population viability. Thus, conservation interventions should safeguard sufficient habitats of high enough quality for survival, successful reproduction to sustain or increase in numbers, and facilitation of dispersal for genetic diversity and (re)colonisation of surrounding habitats (Lawton et al. 2010). Designing resilient ecological networks should therefore take its starting point in how ecological processes respond to changes in habitats’ spatial attributes, based on context-dependent considerations of ecological mechanisms (Fig. 1). For example, processes that strengthen populations locally in the landscape, such as survival and reproduction, are the primary focus of the theory of landscape complementation and supplementation (Dunning et al. 1992), whereas processes that strengthen the relationships between populations at larger landscape scales, such as dispersal, play a larger role in island biogeography (MacArthur and Wilson 1967) and metapopulation theory (Levins 1969; Hanski 1999). An explicit focus on ecological processes and mechanisms would enable predictions of intervention outcomes based on, e.g. process-based models, complemented, or adjusted to a relevant local context by applying knowledge regarding the specific conservation objective, including target species or ecosystem, land-use, threats, management challenges, and stakeholder interactions (Groves et al. 2002; Wilson et al. 2007; Dawson et al. 2011).

Holistic conservation objectives and policies are however often set through strategies and practical considerations that also need to include political and socio-economic aspects, with objectives agreed upon between multiple actors and sectors with potentially conflicting interests (Ludwig et al. 2001). As an example, the European Strategy on GI seeks to enhance biodiversity conservation through the promotion of ecological networks, in addition to supporting healthy ecosystems, sustainable development, mitigation and adaptation to climate change, and to strengthen a wide range of ecosystem services (European Commission 2013). These multiple objectives have, however, led to different implementations of the strategy in different European countries (Slätmo et al. 2019). Some countries have applied the strategy with a direct biodiversity conservation objective aiming to develop resilient and coherent ecological networks for biodiversity and ecosystem services (Salomaa et al. 2017; Slätmo et al. 2019; von Post et al. 2023), while others have applied it in a more general land-use and spatial planning context with only indirect links to biodiversity conservation (Slätmo et al. 2019). In particular, the ecological landscape perspective necessary for conservation of biodiversity is vaguely represented in the European strategy on GI, and prioritisations according to evidence-based understandings are partly missing (von Post et al. 2023).

Notably, within a biodiversity context, GI is often interpreted as having a particular focus on the structural connectivity between habitats (Wright 2011; Garmendia et al. 2016; Salomaa et al. 2017). However, in relation to other objectives the interpretations of GI vary (see Wright 2011; Slätmo et al. 2019). Although connectivity in general terms is an important component in ecological mechanisms operating across landscape mosaics, increasing structural connectivity as such may not primarily contribute to increasing population size or viability (Hodgson et al. 2011), i.e. not directly supporting biodiversity. Hence, the links between GI and efficient biodiversity conservation may actually be relatively weak, which to some extent reflect the fact that GI has multiple goals beyond biodiversity conservation (von Post et al. 2023). Implementing GI may therefore not help solve the problem of decreasing biodiversity, unless trade-offs between other intended outcomes are explicitly considered (von Post et al. 2023).

A relevant ecological landscape perspective that resilient ecological networks (and GI) could be based on has been adopted in ongoing implementations of conservation measures in the UK (Isaac et al. 2018). In a report to the Department of Environment, Food and Rural Affairs (DEFRA), Lawton et al. (2010) suggested a strategy based on four well-established principles for biodiversity conservation (Isaac et al. 2018). The ecological landscape perspective is there based on the concept Better, Bigger, More, and Joined (BBMJ), i.e. conservation interventions should strive to increase habitat quality (Better), habitat size (Bigger), habitat quantity across the wider landscape (More), and finally connectivity (Joined) (Lawton et al. 2010). Combined, these components promote resilient ecological networks for the conservation of multiple species in the long term, while the order BBMJ reflects the separate components’ relative importance for population viability at the landscape scale (Isaac et al. 2018). Habitat features such as patch size, habitat amount, quality, fragmentation, and connectivity, have been shown to affect ecological processes determining population viability in various ways (MacArthur and Wilson 1967; Hanski 1999; Fahrig 2003; Hodgson et al. 2011). The review by Hodgson et al. (2011) suggests that, especially with a changing climate, habitat area and quality are of primary importance through their effect on population size and reproduction, enhancing both long-term persistence of local populations and the production of the propagules that can benefit from enhanced connectivity. Furthermore, functional connectivity, i.e. the connectedness of local populations through movement and dispersal between different parts of a landscape, is expected to increase with an increasing habitat availability and quality across the landscape, whether habitats are structurally connected or not (Hodgson et al. 2011).

Although the theoretical frameworks that BBMJ is based upon are well established, reviews on the relative importance of the individual interventions have reached partly contrasting conclusions (see for instance Hodgson et al. 2009; Doerr et al. 2011; Fahrig 2017; Fletcher et al. 2018). These discrepancies likely arise from the complex relationships between habitat attributes, ecological processes and mechanisms. Biodiversity policies may rely, more or less explicitly, on these different theories and assumptions, which in turn may have consequences on how conservation actions in a GI context are formulated (von Post et al. 2023).

In this study, we argue that there is a lack of explicit consideration of ecological processes within the concept of GI and in research related to GI, whereas such explicit considerations are needed for practical conservation efficiency. We base this argument partly on a previous policy-analysis (von Post et al. 2023), showing that explicit biodiversity prioritisations are missing within EU GI-policy, leading to the risk of biodiversity losing out in favour of other policy interests. Whereas this risk may be relevant also in other contexts, it seems to be particularly so in GI within EU (von Post et al. 2023). In the present study, we explore biodiversity conservation prioritisations within the broader context of GI by first investigating what ecological mechanisms (Fig. 1) that are represented in the scientific literature on GI that explicitly addresses biodiversity conservation objectives, and secondly, by comparing the outcome with empirical evidence on how spatial habitat attributes that underpin the BBMJ-principles affect ecological processes essential for population viability, e.g. reproduction, survival, and dispersal. Based on these two steps we discuss how evidence-based conservation principles diverge or come together in the concept of GI, and how strategies promoting GI or resilient ecological networks could be strengthened to better contribute to biodiversity conservation.

## Ecological processes in research on GI and biodiversity

The GI concept implicitly embraces the notion of bigger, better, more and joined habitats, at least in the context of spatial planning as it was first formulated in the United States (Benedict and McMahon 2006). The roles of the different components that define a GI have been described as “hubs” and “links”, where the hubs, i.e. the habitats or the clusters of habitats, where populations of species live and reproduce, are considered the backbone of the GI, while the links between the hubs facilitate movement among them (Benedict and McMahon 2006). Later, however in European policy context (European Commission 2011, 2013) this dual focus has weakened as the emphasis on the links has become dominant relative to the habitats (hubs), thereby emphasising ecological processes tied to species movement and dispersal (Garmendia et al. 2016; Salomaa et al. 2017; von Post et al. 2023). Even if protected, large areas (i.e. Natura 2000 areas) are still considered the backbone of the European GI network (European Commission 2013), this shift in focus may reflect an increasing tendency to consider GI as a tool for climate change adaptation, where facilitating species’ dispersal over larger spatial scales becomes an important conservation solution (Hodgson et al. 2016; von Post et al. 2023).

The scientific literature on GI, in turn, spans across several disciplines, covering topics such as sustainable development, urban spatial planning, biodiversity conservation, and ecosystem services (Chatzimentor et al. 2020; Ying et al. 2022). Because of this disciplinary variability, it is not apparent to what extent research on the GI concept actually considers ecological processes relevant for biodiversity conservation.

In a report to the environmental protection agency in Sweden on the effects of GI on biodiversity (Ekroos et al. 2020), we conducted an adapted systematic review to investigate the extent to which the scientific literature on GI in relation to biodiversity explicitly refers to relevant ecological mechanisms underpinning resilient ecological networks. The literature search and the following review process captured 211 relevant scientific articles that included aspects of biodiversity or biodiversity-related ecosystem services (pollination and pest control) (searches conducted in November 2019, see Supplementary Information S1 and Ekroos et al. 2020). We conducted a wordcount on the identified articles using a set of terms closely associated with the ecological mechanisms of relevance; connectivity, metapopulation, source-sink, landscape supplementation and complementation, and spill-over (see Fig. 1 for an overview of the theoretical frameworks that address these mechanisms and how they relate to ecological processes relevant for population viability). For this study we also conducted a wordcount (including their reference lists) using terms of relevant ecological processes addressed in the theoretical frameworks; reproduction, survival, mortality, dispersal, immigration, emigration, colonisation, and extinction. The wordcount revealed that a vast majority of references were made to connectivity (in 64% of the articles, Table 1) and with most references to dispersal processes (in 40% of the articles, Table 1), even if addressed processes were more balanced (extinction 35%, colonisation 27%, survival 26%, and reproduction 24%, see Table 1). The result indicates that research on GI and biodiversity to a higher extent engages with the concept of connectivity, over and above ecological mechanisms that more explicitly determine population viability (Fig. 1). Although a connectivity framing as such does not exclude the possibility to integrate habitat quality, quantity and configuration in modelling approaches (Wood et al. 2022), our results show that research on GI and biodiversity rarely make explicit links to relevant theoretical concepts relevant for biodiversity conservation in mosaic landscapes.

**Table 1:** Results from the wordcount to investigate what ecological mechanisms (terms) that are referred to in the scientific literature on Green Infrastructure that includes a focus on biodiversity, and what ecological processes that are considered. The wordcount was done separately for the number of articles examined (211 in total), and the number of times that the terms were referred to, showing a strong focus on connectivity relative to other ecological mechanisms, and a slight dominance of dispersal-related processes

Term	No articles	No of times
*Mechanistic frameworks*		
Connectivity	136	2438
Functional connectivity	35	173
Metapopulation	34	87
Island biogeography	11	16
Source-sink	6	6
Landscape supplementation	1	1
Landscape complementation	1	1
Spill over	0	0
*Ecological processes*		
Dispersal	84	502
Extinction	75	147
Colonisation/colonization	58	156
Survival	54	132
Reproduction	50	92
Mortality	35	97
Immigration	13	30
Emigration	0	0

**Fig. 2:**
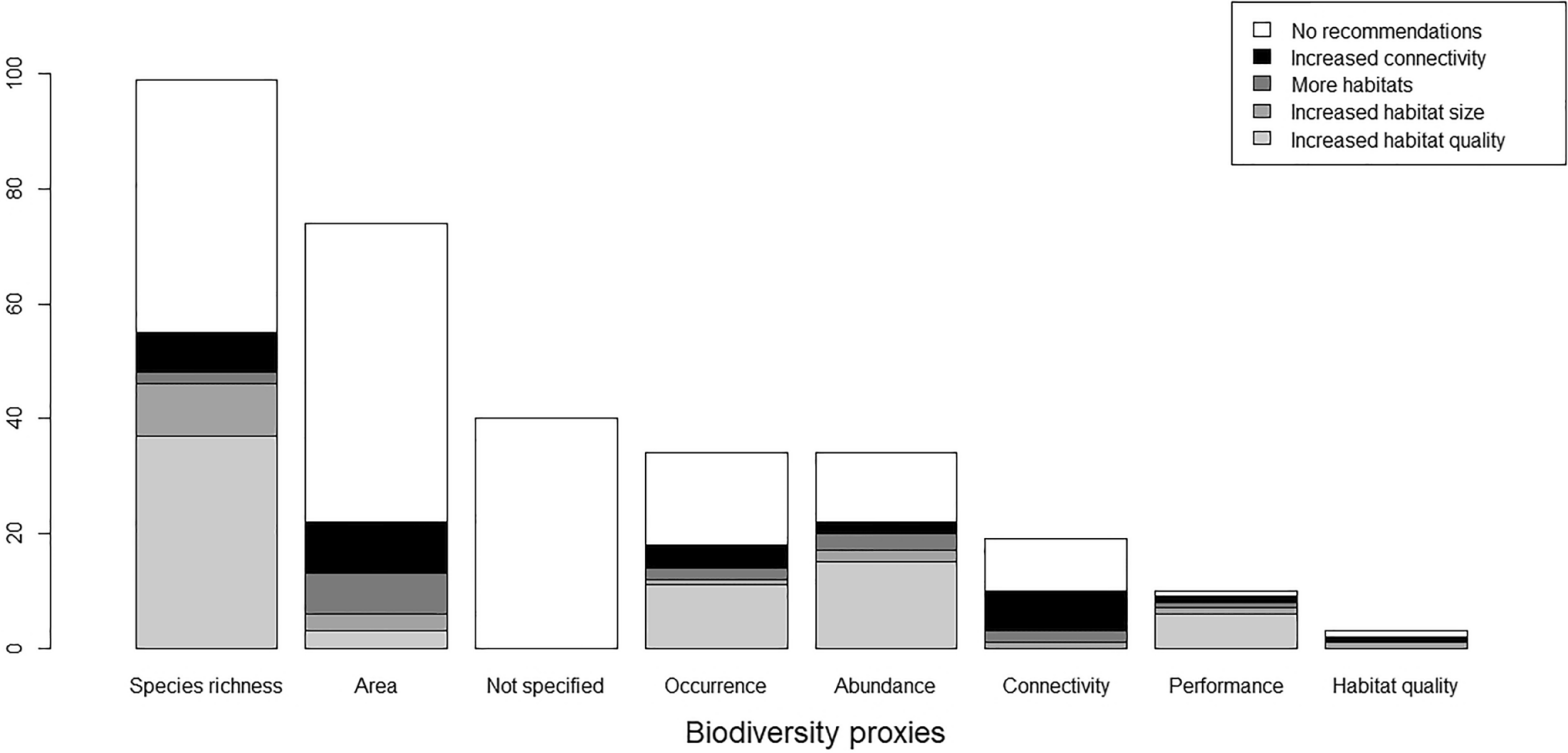
Results from the content analysis showing what biodiversity proxies that are considered and/or measured within the scientific literature on green infrastructure that includes biodiversity conservation framing. The legend shows the proportionate conclusions drawn regarding recommended actions to promote biodiversity

A second finding in our review (Ekroos et al. 2020) was that the type of biodiversity measures used in the scientific GI literature to a large extent were based on implicit biodiversity proxies (43% of all 313 cases identified in the 211 articles; Fig. 2), such as habitat area (24% of cases), presence of green links (6% of all cases), or unspecified biodiversity (13% of cases). Thus, almost a third of all studies on GI and biodiversity use different kinds of land-use or habitat areas as proxies for biodiversity. This can be justified, e.g. when assuming a species-area relationship (Pereira and Daily 2006; Olsson et al. 2021) but remains a very coarse biodiversity measure that does not in any way inform us about how GI affects the underlying ecological processes that ultimately determine conservation outcomes. Considering that the selection of scientific studies on GI targeted those including biodiversity aspects, this seemed somewhat surprising, although the patterns of biodiversity that these indirect measures present could be further explored to better understand the ecological processes.

More explicit measures of biodiversity may better reflect the value of GI for biodiversity conservation. While the majority of studies on GI and biodiversity do use explicit biodiversity measures (57% of all cases identified), these are dominated by studies focusing on species richness (32% of all cases), which is a criticised biodiversity metric because it gives little or no information about population viability of the species present in a given system (Phalan et al. 2011; Hanski 2015, but see Olsson et al. 2021). Biodiversity measures based on aggregated species-specific abundance relationships may be most informative regarding conservation outcomes under alternative land-use strategies (Phalan et al. 2011), because they enable estimations of densities of species in different contexts. However, metrics such as abundance or presence suffer from the potential problem that local populations may be dependent on immigration from other (source) habitats (Pulliam 1988, see Fig. 1). Measures of demographic parameters would be most informative to assess biodiversity outcomes, but obtaining such information is laborious and only a few cases have used such measures in the context of GI (3% of all cases). In those cases, the majority suggest increasing local habitat quality to be most important for biodiversity conservation, which is consistent with BBMJ principles for resilient ecological networks. However, most studies in the scientific literature addressing GI and biodiversity using species richness or abundances, or implicit biodiversity measures, did not suggest specific conservation actions or prioritisations (see Fig. 2).

In summary, we argue that the ecological processes that in fact determine population viability and long-term persistence are poorly integrated in the scientific literature on GI. First, if ecological processes are mentioned or described at all in the GI scientific literature, these are biased towards connectivity, and explicitly or implicitly, address dispersal processes more often than other processes directly determining population viability. In spatial planning and in policy (von Post et al. 2023), as well as in research (Hodgson et al. 2011), connectivity may thus become overemphasised in relation to other habitat features important for ecological processes that matter for long-term conservation of populations. Secondly, empirical research on GI is to a relatively large extent relying on fairly uninformative biodiversity metrics from a conservation perspective. Only a few studies considered demographic parameters, which would be most informative regarding conservation outcomes of alternative land-use decisions.

## Ecological processes in landscape mosaics—the empirical evidence

The strong conceptual focus on connectivity within GI may, perhaps inadvertently, be contradictory to the BBMJ principles when connectivity is conceptualised without clear links to other ecological mechanisms relevant for resilient ecological networks (see above). This is important because connectivity can have different effects on ecological processes determining population viability (Hanski 1999). As an example, concerning relatively small spatial scales, connectivity between nesting and feeding sites is important for the reproduction and survival of central-place foragers such as birds and bees that use different habitats for nesting and foraging (Olsson and Bolin 2014). Across larger spatial scales in turn, metapopulation or source-sink dynamics rely on dispersal between reproductive sites that may be important to sustain reproductive local populations (Hanski and Ovaskainen 2003). Under ongoing global change, dispersal barriers may become strengthened or weakened, with potential consequences for regional population persistence or range shifts (Caplat et al. 2016). However, in all these cases, the availability of key habitats is more important than connectivity per se, although connectivity and habitat availability are also intrinsically interlinked (Moilanen and Hanski 2001). Hence, in a GI context, it will be important to understand the extent to which different types of habitat and landscape features affect ecological processes that underpin population viability in specific cases.

To illustrate how different habitat and landscape features affect survival, reproduction, and dispersal, we mapped the empirical evidence of the underlying ecological mechanisms important for the creation of resilient ecological networks, using an adapted systematic review process described by Luederitz et al. 2016 (see S1 for schematic description of the different review steps and a full description of method, analysis, and detailed results). This method allowed us to quantitatively summarise scientific studies and results on effects from different habitat and landscape features explicitly on ecological processes, focusing on terrestrial environments. We used the following search strings in combination to identify relevant scientific papers, using two different databases on scientific articles, Web of Science (BIOSIS, CABI, Zoological, Medline and Core collection) and Scopus:

*Habitat and landscape features of interest*; (“habitat area*” OR “habitat quantit*” OR “habitat connectivit*” OR “habitat isolation” OR “habitat structure*” OR “habitat aggregation*” OR “aggregated habitat*” OR “habitat fragmentation*” OR “fragmented habitat*” OR “habitat configuration*” OR “habitat amount*”) NOT TS = marine NOT TS = fish.

*Ecological processes of interest*; (productivity OR mortality OR survival OR reproduct* OR dispersal OR immigration* OR emigration* OR extinction* OR colonisation* OR colonisation* OR recolonisation* OR “recolonisation*” OR “recolonisation*” OR “recolonisation*” OR “population demograph*”) NOT TS = marine NOT TS = fish.

Note that we did not include “habitat quality” as a search term, because here we intentionally focused on spatial aspects that define the landscape structure. However, we noted the effect of habitat quality amongst the studies that also explicitly considered at least one of the above terms defining landscape structure. Further, we only included direct measures of the ecological processes, and therefore we did not include indirect measures such as abundance or population size. Thus, we were interested in what and how the underlying ecological mechanisms respond to changes in habitat and landscape structural features. We identified in total 10 973 unique articles from searches in Web of science and Scopus (May 2018) (see process chart S1, Fig. S2 for further details regarding review steps and specific numbers of studies excluded after each step). Due to limited time and resources, we scanned a subset of 5000 (initially 1373 in alphabetical order and later 3627 in a randomised order) articles based on title and abstract using three main decision criteria and two support questions (see details in S1). After sequential exclusion steps, 1637 results (cases) from 342 unique articles remained for the summary, including results on population level effects from habitat and landscape features. Since ecological processes were studied using a large variation of response variables, these were recategorised into three main and nine sub-categories (see S1 for detailed description); *population persistence*, including extinction, mortality, and survival, *productivity processes*, including measures of reproduction and growth, and *movement processes*, including dispersal, colonisation, emigration, and immigration. We processed and illustrated the extracted information using R version 4.0.5 (R Core team 2021), library networkD3 (Allaire et al. 2017).

**Fig. 3:**
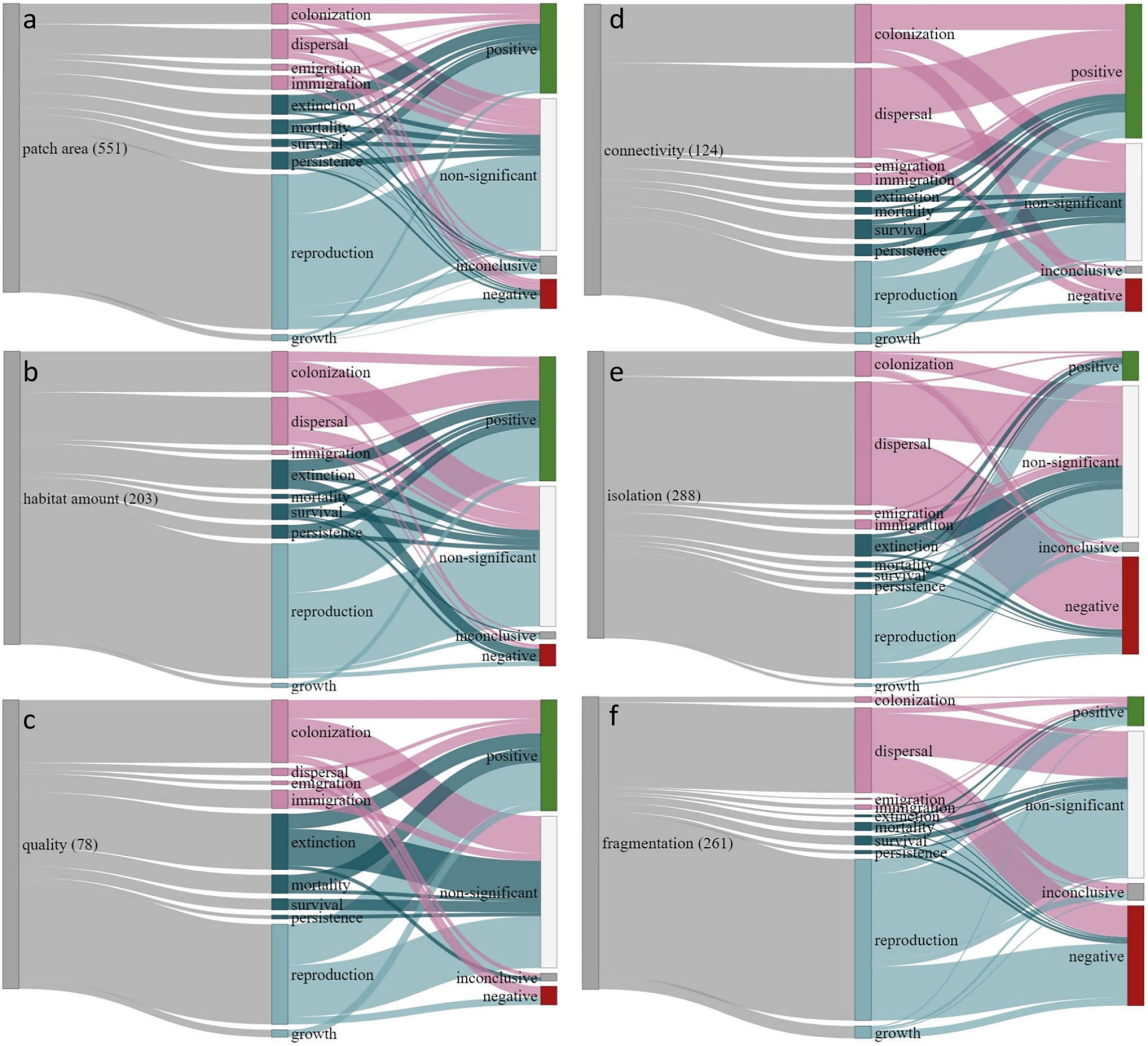
The proportions of results and the total number of cases identified for the habitat features patch area (**a**), habitat amount (**b**), habitat quality (**c**), connectivity (**d**), isolation (**e**), and fragmentation (**f**). Ecological processes are classified in three different types, represented with separate colours: movement processes in pink, population persistence in dark turquoise, and productivity processes in light turquoise. Increasing habitat patch area (**a**), amount (**b**) and connectivity (**d**) show predominantly positive effects on ecological processes important for population viability, e.g. reproduction, while isolation (**e**) and fragmentation (**f**) show predominantly negative effects. Connectivity as such has to a relatively lower extent been studied in relation to reproduction, and with mixed results. Overall, a majority of the studies could not establish a clear link between the studied habitat features and ecological process. The effects on extinction and mortality were reversely included to correspond to the positive net effect on population viability

First, our review shows that ecological processes of reproduction, dispersal, and colonisation are predominantly positively influenced by increasing patch area, habitat amount, and habitat quality (Fig. 3a-c), also leading to reduced extinction, which is according to expectations. Similarly, dispersal, immigration, colonisation, growth, and reproduction are to the most part positively affected by connectivity (Fig. 3c). On average, our review also showed reduced dispersal and colonisation, lower reproduction and population growth, from increasing isolation and fragmentation (Fig. 3e, f). Notably, a majority of studies did not show a significant effect on the ecological processes from the habitat features studied (Fig. 3). This was the case for all habitat features, but in particular for those describing habitat shape or configuration, which also showed more equal proportions of positive and negative effects on the ecological processes studied (see S1).

Secondly, the most studied ecological process was reproduction (nearly 50% of all 1637 cases in total), followed by fewer cases for dispersal, colonisation, and extinction, and a limited number of cases for other ecological processes (less than 50 cases in each process category; Fig. 3). Reproduction was most commonly related to attributes describing patch area, followed by fragmentation and habitat amount (Fig. 3a). Overall, patch area was the most studied habitat feature (one third of all cases), followed by isolation, fragmentation, and habitat amount, whereas other features were studied to a lesser extent (Fig. 3, see S1 for details).

**Fig. 4:**
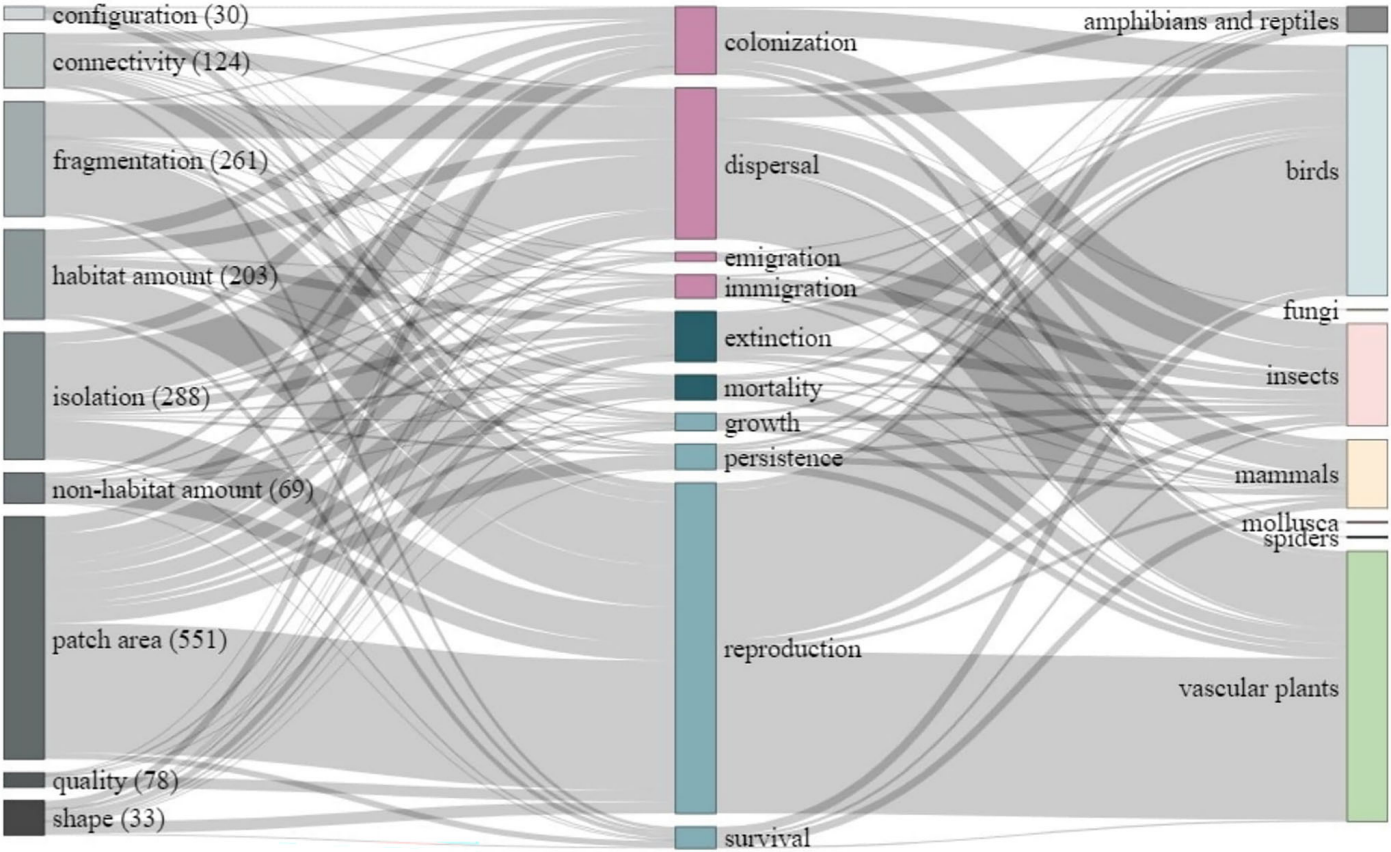
Distribution of proportions of identified cases (in total 1637 cases from 342 unique studies) and studied relationships between habitat features (left column), ecological processes (in the middle), and taxonomic group (right column)

Thirdly, our review shows that ecological processes linked to population viability are relatively less well studied in relation to connectivity as such (see Fig. 4). Positive effects of connectivity on reproduction are mainly driven by studies on birds and plants (Fig. 4), which would be consistent with mechanisms such as landscape complementation and foraging connectivity (Ekroos et al. 2016), or pollinator availability (Hederström et al. 2024). In contrast, connectivity or isolation, both associated with the spatial arrangement of habitats, were studied more often in relation to movement processes (Fig. 4). Overall, dispersal was the second most studied process, and it was most commonly related to attributes describing habitat isolation, followed by fragmentation, and thereafter by patch area. Dispersal, habitat isolation and fragmentation are conceptually closely related to connectivity in a metapopulation context (Hanski 1999), and the framings in these studies are consistent with the concept of landscape connectivity, which by definition emphasises how the landscape structure itself affects how organisms disperse across a given landscape (Kindlmann and Burel 2008). Only 8% of the cases of studied relationships focused on effects of connectivity, and relatively few studies have assessed effects of connectivity on ecological processes most important for population viability, such as reproductive success, which is also the case concerning the scientific literature on GI reviewed above. We note that considering abundance as an indirect measure of ecological processes (the balance between reproduction, mortality, and migration) would likely have resulted in more studies related to connectivity, but such studies would be less able to determine the underlying mechanisms behind the patterns (c.f. Fischer et al. 2014).

In summary, we show that reproduction and survival, two important processes for determining population viability, are less often strengthened by increasing connectivity compared to increasing patch area and habitat amount, which seems to contrast to the dominant role of connectivity in the framing of the scientific GI literature (Table 1). Although the intention of our review was not primarily to demonstrate the relative effects of particular habitat features on ecological processes, the results are consistent with the argument that resilient ecological networks should be based on the BBMJ principles to prioritise better quality habitats of larger size, over better joined habitats, for long-term conservation of biodiversity and populations. Although connectivity plays an important role across a variety of spatial contexts, as discussed above, we argue that there is a risk that generic recommendations to enhance biodiversity conservation by increasing connectivity may become misleading by being oversimplified and downplaying other important actions.

## Conclusions

Despite a huge variability in mechanistic responses, and a clear bias regarding relationships and taxonomic groups studied, interventions that enhance connectivity in a GI-framework for biodiversity conservation should not be favoured at the expense of maintaining large enough habitats with high quality. We argue that the current lack of clear biodiversity objectives represented in the EU GI policy (von Post et al. 2023), and the imprecise connectivity framing for the development of resilient ecological networks represented in the scientific literature on GI, contributes to vague conservation goals. Whereas there is nothing inherently wrong with strengthening connectivity (see e.g. Isaacs et al. 2018), directing more focus on connectivity rather than on habitat area and habitat quality may be ineffective from a biodiversity conservation perspective, as we know that habitat loss and degradation is one of the main causes for biodiversity loss (IPBES 2019). Interventions improving connectivity will, however, be important in cases where strong dispersal barriers threaten populations of conservation concern (Arponen et al. 2013; Caplat et al. 2016; Isaac et al. 2018), or, e.g. when considering optimal sites to increase floral resources for pollinators that have characteristic foraging distances (Olsson et al. 2015). In addition, the connectivity concept can be useful as a pedagogical tool to highlight that habitats and organisms are spatially and temporally connected through ecological processes and drivers that take place across entire landscapes.

It has been suggested that the broad definition of the GI concept is beneficial, as it can bring together a diverse set of actors to find common solutions (Garmendia et al. 2016). However, at the same time the heterogeneity in how GI is understood and interpreted may become problematic from a biodiversity conservation perspective because, as we have shown here, it may direct focus and efforts towards less effective interventions. We argue that GI policies aiming to safeguard biodiversity must more explicitly consider ecological processes necessary for population persistence. Whilst this is not a new idea in a general sense (Moilanen and Hanski 2001; Hodgson et al. 2011), an inherently multifunctional GI (or resilient ecological networks) policy framework needs to build on clear spatial prioritisations in terms of where to target broader environmental outcomes such as ecosystem services, and where to target biodiversity conservation as such, and in the latter case explicitly consider all ecological processes that enhance population viability in a landscape perspective.

## Supplementary Information

Below is the link to the electronic supplementary material.

Supplementary file1 (PDF 2250 KB)
