# Supplementary material for: Green infrastructure has weak conceptual links with efficient biodiversity conservation
Source: Ambio. 2025 Feb 22;54(7):1153–64. doi: 10.1007/s13280-025-02149-1 (PMC12133648; doi:10.1007/s13280-025-02149-1)
Supplement: Supplementary file 1 — Supplementary file1 (PDF 2250 KB) [file 13280_2025_2149_MOESM1_ESM.pdf]

Ambio

Supplementary Information

This supplementary information has not been peer reviewed.

Title: **Green infrastructure has weak conceptual links with efficient biodiversity conservation**

Johan Ekroos[2,3,4,**], Maria von Post[1,2*,**], Anna S Persson[2], Martin Stjernman[1], Ola Olsson[1]

Affiliations:

[1]Department of Biology, Lund University, Lund, Sweden

[2]Centre for Environmental and Climate Science, Lund University, Lund, Sweden

[3]Department of Agricultural Sciences, University of Helsinki, Helsinki, Finland

[4]Helsinki Institute of Sustainability Science, HELSUS, University of Helsinki, Helsinki, Finland

*Corresponding author

**J Ekroos and M von Post share first authorship

Contact Maria von Post
e-mail: maria.von_post@biol.lu.se

Methods description and detailed results from the review on ecological perspectives in green infrastructure scientific literature and of habitat features effects on ecological processes.

Biodiversity and ecological processes in scientific publications on green infrastructure

The review was conducted as a part of an assignment to the Swedish government and has been previously published as a report in Swedish by the national environmental agency (EPA) (Ekroos et al. 2020). The purpose of the supplementary description is to give full transparency to the original methodological process and results reported in the main text.

Material and Method

To identify and summarize relevant scientific literature on effects on biodiversity from green infrastructure we conducted an adapted systematic review process (Luederitz et al. 2016; see Figure S1 for schematic description of the different review steps and A1 for metadata extracted in the review process). We used two search terms to identify relevant literature, “green infrastructure” and “biodiversity”, and ran the search in two different databases of scientific literature, Web of Science Core collection and SCOPUS. The results were thereafter merged, and duplicates were removed. Identified articles were scanned in two steps and included or excluded based on a predefined set of criteria suitable for identifying studies relevant for effects on biodiversity. We first scanned the title and abstract using simple criteria to only include studies with a clear focus on effects on biodiversity and/or ecosystem services linked to biodiversity, e.g., pollination or pest control. To fit the assignment set out by the EPA, studies included in the review should be relevant for temperate regions and restricted to terrestrial environments.

In the second step we scanned the full text of the included studies from step one and extracted relevant information to map and describe effects of green infrastructure on biodiversity. Information extracted from relevant articles covered the following seven categories; 1) main focus on biodiversity, or on biodiversity-related ecosystem services; 2) type of land-use scenario (urban, forest, aquatic, mosaic landscapes); 3) how green infrastructure was defined (explicit physical structure and type of structure, or abstract, implicit definition); 4) definition and used measures of biodiversity; 5) conservation recommendation conclusions; 6) information on whether the study was empirical, theoretical or a review; and 7) if the study referred to theoretical frameworks relevant for spatial conservation planning or implementing green infrastructure interventions (island biogeography, metapopulation, connectivity etc.). During the full text process further studies were excluded if it was revealed that the focus was not in line with the criteria in the initial title and abstract scanning, or if a full text document was missing (see Appendix S1 for metadata extracted in the review process).

Initially we identified 636 unique studies (November 2019). After the two sequential review steps, 211 unique studies remained for the summary.

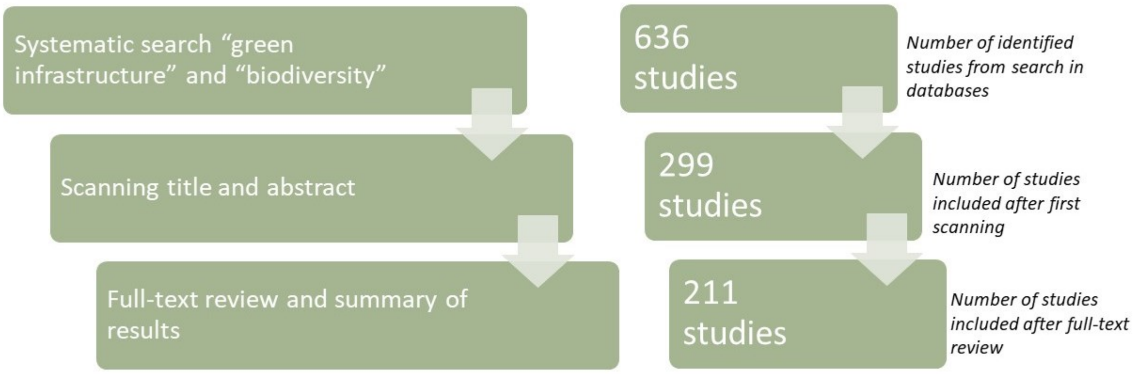

Figure S1. Schematic description of the review steps and number of identified studies on green infrastructure and biodiversity, figure adapted from Ekroos et al. 2020.

Analysis

We processed and illustrated the results using R version 4.0.5 (R Core team 2021). The extracted information was first restructured to facilitate summation on green infrastructure effects on biodiversity and associated ecosystem services (see Table S1). We defined main categories for the extracted information on; in what environment green infrastructures were studied, what type of green infrastructure that was studied, what biological proxies that were used to measure the effects, and if conclusions regarding conservation recommendations were present (see Table S1 for short version of reclassifications relevant for this study and Ekroos et al. 2020 for the full version of the table). The results were thereafter summarized and illustrated using two different levels, including a general, overall analysis based on number of studies, and a more detailed analysis using number of cases in total, as some studies included multiple aspects of green infrastructure and biodiversity.

Table S1. Reclassification into main categories of extracted information on green infrastructure effects on biodiversity

Main category	*Sub-category*	*Extraced terms from the review*
**Landscape type**	*Not specified*	all land-use types, non-specified land use
	Forest	wood land
	Aquatic	water area, coastal water
	Mosaic landscapes	cropland/artificial land, cropland/woodland, cropland/woodland/artificial land, wetland/grassland, woodland/artificial
**Type of green infrastructure**	*Urban green spaces*	flowering lawns, urban residential areas, urban woodland, urban cemeteries, vacant lots, urban parks, Right of Way Bioswale, Vegetated swale, urban forest, urban meadows, urban planting, urban orchard, urban vacant lots, Urban Green spaces bioswale soil, urban grassland, brownfields, urban allotments, green walls, urban trees, green roofs, suburban green spaces, Urban green spaces, urban green elements, community gardens, domestic gardens, easement gardens
	Linear landscape elements	greenway, Linear transportation Infrastructures, roadside vegetation, railway, urban linear infrastructure, linear road elements, linear landscape elements, linear grassland elements, hedgerow, road alley
	Blue-green	flood-control basins, channelized watercourses, drainage pumping stations, remnant ponds, floodplain, foreshore constructions, river flood-plain, riparian, rock pool, structures to manage storm water, urban blue and green elements, Urban Drainage System Elements, urban lake, urban watershed, wetland, riparian forest, urban green spaces/inland water, artificial wetland, Artificial Floating Islands, catchment area, river
	Connectivity	urban green space connectivity, network, connectivity, least cost path, functional connectivity, corridor, hubs and links,
	Forest	forest, riparian forest, deciduous forest, forest massifs, woodland, semi-natural vegetation, urban forest
	Others	European green belt, treed common, unmanaged grassland, protected areas, recreational sites, post-mining sites, brown fields, Natura 2000, grassland, wetland/inland water/grassland/forest, semi-natural areas, Urban and peri-urban agriculture, alpine meadows/slopes
**Biodiversity proxies**	*Species richness*	species diversity, species richness, species composition, species assemblages, Shannon diversity, diversity index, number of threatened species, biodiversity, species classification
	Presence or abundance	species occurrence, occurrence, presence, distribution, species distributions, placement, occupancy, species abundance, activity, biomass, population size, density
	Demographic measures	extinction risk, trend, tree browsing damage, damage level, performance, functionality, growing season, survivorship
	Area	area, length, amount, fragmentation, cover
	Green links	habitat connectivity, connectivity
	Habitat quality	quality, heterogeneity
	Not specified	area-connectivity, habitat, species

To further explore the material in the report (Ekroos et al. 2020), we conducted a wordcount to determine to what extent relevant ecological theoretical frameworks and ecological processes were used in the identified scientific articles, using the wordcount function in Adobe Acrobat Reader version 2020 (version 2020.005.30467). We specifically searched for

terms linked to ecological mechanisms of importance in a spatial conservation context, linked to the ecological mechanisms presented in Figure 1 in the main manuscript. The terms searched in relation to ecological mechanisms were “connectivity”, “functional connectivity”, “metapopulation”, “source-sink”, “landscape complementation”, “landscape supplementation”, and “spill-over”. We used short versions of the terms followed by an asterisk to allow a wider detection of the words. The terms searched in relation to ecological processes were “reproduction”, “survival”, “mortality”, “dispersal”, “immigration”, “emigration”, “colonisation OR colonization”, and “extinction.

Results

Out of the 211 identified studies (see Appendix S2 for all included publications) that investigated effects from green infrastructure on biodiversity or associated ecosystem services, 48% had a main focus on biodiversity, and approximately equal share of studies focussed on both biodiversity and ecosystem services (46%). Out of the 98 studies with double focus, 23% included biodiversity associated ecosystem services, i.e., ecosystem services specifically linked to some taxonomic groups that are often also used to assess biodiversity, e.g., pollination or pest control. A few publications had a main focus on ecosystem services only (3%), and one study was not possible to classify due to an explicit focus on multifunctionality.

The identified studies were conducted within different land-use contexts; 60% of the studies, and about the same amount of all cases of results (58%) were conducted within urban environments; 20% of the studies (22% of all cases) were vague or did not specify focus considering landscape types; while other landscape types were less well represented (agricultural landscapes 7% of the studies, forest landscapes 6% of the studies, with both approximately the same proportions of all cases).

Most of the studies focused on explicit, physical types of green infrastructure (94% of the studies). After reclassification into main categories of structures, urban green infrastructures dominated the results (44% of all studies), followed by forest elements (11% of all studies), blue-green structures (9% of all studies), linear elements (7% of all studies), general structures (6% of all studies), and connectivity explicitly (5% of all studies). 33 studies (16%) did not specify the physical form of the green infrastructure studied.

Biodiversity was referred to in various ways and with a variety of proxies. Most of the proxies consisted of measures of species richness (32% of all cases), followed by habitat area (24% of all cases), and 11% of the cases respectively used presence or abundance of species as a proxy for biodiversity. Less common proxies were connectivity (6% of all cases), demographic measures (3% of all cases), and habitat quality (1% of all cases). In 13% of all cases biodiversity proxies were vague and not further specified.

In 32% of all the 211 publications, or in 44% of all of the 313 cases, the authors drew conclusions regarding recommended conservation interventions. Most cases recommended improved habitat quality (23% of all cases), while 10% of the cases recommended improved connectivity, 6% increased areas, and 5% increased habitat amount. Improved habitat quality was more often recommended in those cases where species measures were used as a biodiversity proxy while increased connectivity was more common when using habitat as proxy.

Our additional wordcount to determine the usage of theoretical frameworks relevant for conservation spatial planning showed that most of the studies included references to connectivity (64%), with fewer specifying this to be functional connectivity (16% of the studies). References to metapopulation theory occurred in 18% of the studies while other theories, e.g., island biogeography, source-sink theory, and landscape complementation theory, were referred to in few or no cases. Our wordcount to determine the ecological processes addressed in the literature showed a slight dominance of references to dispersal (40% of the publications), followed by extinction (35%), colonisation (27%), survival (26%), reproduction (24%), and mortality (17%). Immigration and emigration were referred to in few or no publications.

Habitat features effect on ecological processes

Material and Method

We used and modified a systematic review process (Luederitz et al. 2016; see Figure S2 for a schematic description of the different review steps) to quantitively summarize scientific studies and results on effects on ecological processes from habitat attributes. As a first step we decided on relevant search strings to use for identification of articles for the review. We aimed to identify articles that included effects on ecological processes relevant for population persistence, hence the first search string included common terms for demographic and dispersal processes. As we were interested in spatially specific habitat attributes relevant in a

landscape planning perspective the second search string specified commonly used habitat descriptors. Our focus for this review was terrestrial environments and therefore we narrowed our search by including exclusion terms for marine environments and taxa related to these environments. The search strings used in combination were:

1. *For ecological processes of interest*; (productivity OR mortality OR survival OR reproduct* OR dispersal OR immigration* OR emigration* OR extinction* OR colonisation* OR colonization* OR recolonisation* OR "re-colonisation*" OR "recolonization*" OR "re-colonization*" OR "population demograph*") NOT TS=marine NOT TS=fish
2. *Habitat features of interest*; ("habitat area*" OR "habitat quantit*" OR "habitat connectivity*" OR "habitat isolation" OR "habitat structure*" OR "habitat aggregation*" OR "aggregated habitat*" OR "habitat fragmentation*" OR "fragmented habitat*" OR "habitat configuration*" OR "habitat amount*") NOT TS=marine NOT TS=fish.

We identified relevant scientific articles through searches in two different databases on scientific articles, Web of Science (BIOSIS, CABI, Zoological, Medline & Core collection) and Scopus. We limited our search to only include publications classified in the databases as original articles or reviews, and language type English. Reviews were included in the initial search since they are relevant for the framing and the discussion of our results, but they were excluded from the empirical data used for the analysis later in the process. We combined the results from the searches in the two databases into one dataset in Clarviate EndNote 20.3 and exported this into a spreadsheet. Duplicate removal was made in two steps, first in endnote and later double checked and revised in the spreadsheet. After the process of identifying potentially relevant articles, we set up our table of criteria for inclusion or exclusion of the identified articles for the following analysis (see Table S2 for the full table of criteria). The inclusion criteria table consisted of three main decision questions and two additional decision support questions (see Table S2 and Appendix S3 for additional extracted metadata).

Based on the defined criteria, we classified the articles into three categories by scanning the articles title and abstract; include, exclude or maybe regarding processing for the next step, a full text review and detailed information extraction. Only articles classified as "include", e.g., where all five decision criteria for inclusion to review process were fulfilled, were processed in the full text analysis. Initially, we reviewed the identified articles in alphabetical order but

due to the large number of identified papers and limited resources this process was revised into reviewing a subset of randomized articles.

We downloaded all full texts of included articles to an EndNote library and reviewed them for extraction of detailed information regarding the type of study/data (experimental, empirical, theoretical), results (response variables in relation to ecological process, habitat attribute, and direction of effect), habitat types, and taxonomic group. During the full text review further articles were excluded due to lack of specific information related to the initial decision criteria of spatially explicit habitat attributes and population level effects, due to missing empirical data, if articles did not clearly state the significance of the relationships based on p-value information or based on AIC, or due to missing full text document. All extracted information was entered into a spreadsheet.

Table S2. The three main decision criteria and the two sequential support questions used in the review process to identify relevant articles for further full text processing.

Inclusion criteria (yes/no/maybe)	***Explanation***
Ecological process	*Does the article relate to ecological and demographic processes at species/population level, excluding biodiversity patterns at community level (diversity/species richness)?*
Habitat attribute	*Does the article relate results on ecological process to habitat attributes defined in search string; habitat area, quantity, connectivity, isolation, structure, aggregation, fragmentation, configuration, amount. Quality is only relevant in combination with other attributes, not quality within a habitat.*
Terrestrial landscape planning context	*Is the article relevant in a terrestrial landscape context as defined by this project; at least partly terrestrial, above ground, mainland, and studies only interesting in relation to a specified spatial context*
Additional support questions	
Scales - Spatial conservation planning context (too small/relevant/too big/not relevant)	*Is the scale relevant in a landscape planning perspective? Too small scales acting within habitat patches is not relevant if not in relation to other habitats. Too big continental scale is not relevant due to the interest in regional conservation planning context. Time scale is also considered here,*

	historical patterns excluded. Not relevant refers to studies where the scales are not defined to determine relevance for spatial conservation planning.
Relevant for the review process or relevant for the overall picture (review/discussion)	*Is the article relevant for the summary of results or for the framing and discussion? Only original papers including at least partly empirical data is relevant for the summary of results.*

We identified in total 10 973 unique articles from searches in Web of science and Scopus (May 2018) (see process chart in Figure S2 for further details regarding review steps and specific numbers of studies excluded after each step). We scanned a subset of 5000 (initially 1373 in alphabetical order and later 3627 in a randomized order) articles based on title and abstract using our three main decision criteria and two support questions specified above (Table 2). After sequential exclusion steps described in the methods and in Figure S2, 1637 results (cases) from 342 unique articles remained for the analysis (see Appendix S4 for all included publications sorted by relevant ecological process).

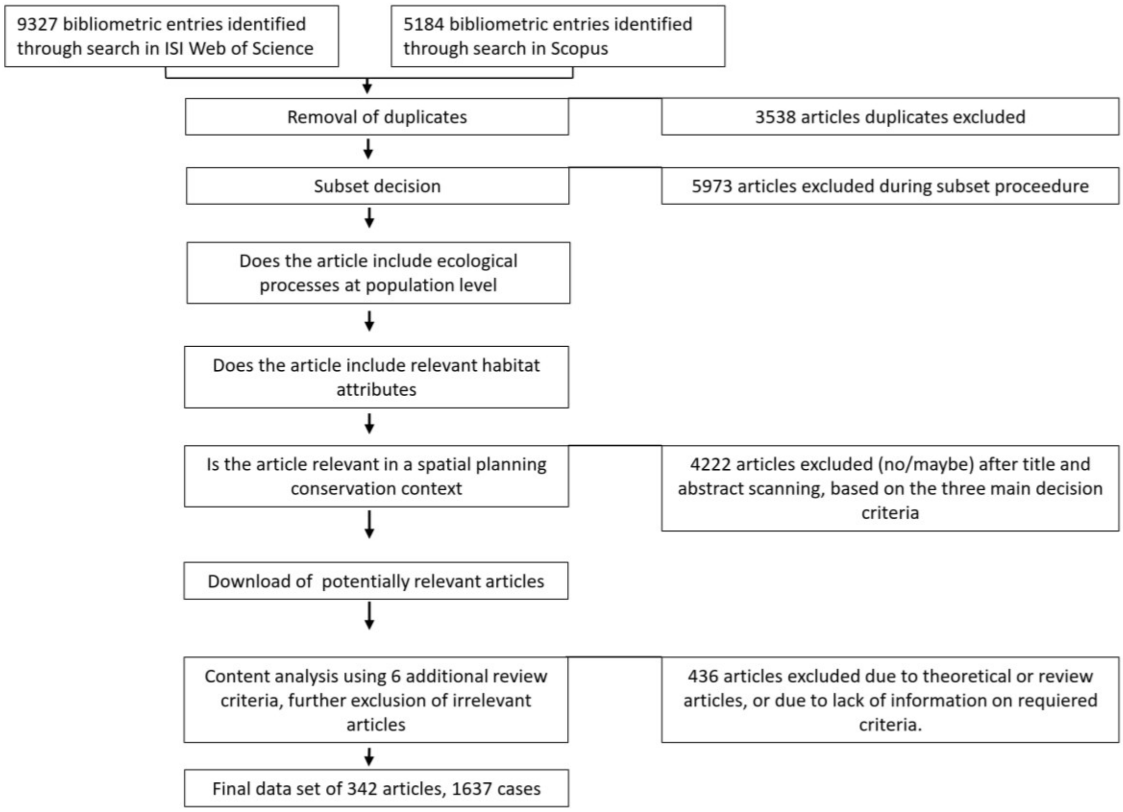

Figure S2. Schematic description of the review steps with number of articles excluded in each step and number of articles and unique case results included in the summary and analysis.

Analysis

We processed and illustrated the extracted information using R version 4.0.5 (R Core team 2021). For illustrations of the relationships found between habitat attributes, ecological processes, and taxonomic groups we used library networkD3 (Allaire et al. 2017). Initially, we homogenized the terminology from the extracted information to facilitate summation. First, we re-classified the variables into main categories since the literature was characterized by a highly varied terminology (see Table S3 for an overview of re-classifications). To this end, we re-classified habitat attributes into the following nine main categories: fragmentation, habitat amount, patch area, connectivity, isolation, non-habitat amount, configuration, shape, and quality. The main categorization type was used for illustrating the results. In a similar vein, we re-classified ecological processes into nine categories, based on the response variables and conclusions described by the authors: colonization, emigration, immigration, dispersal, mortality, extinction, population growth, population persistence, reproduction, and survival.

Table S3. Reclassification of habitat features and ecological processes into main categories for summations.

Main category	***Sub-category***	***Direction of effects on population viability***	***Examples of terms***	***Number of unique terms***	***Description***
**Habitat features**					
Patch area			area, size	25	Expressions of defined patch/habitat size on patch scale
Habitat amount			proportions, number of patches, patch density, habitat cover	55	Measures of habitat within a given area
Non-habitat amount			proportions of unfavorable habitat	17	Measures of non-preferred habitat by the organism within a given area
Connectivity			indices, proximity measures, number of connections, density of habitats	29	Measures of distances, indices, or amount of habitat within buffers expressed as connectivity by author
Isolation			distance to/from, isolation indices, gap size	62	Measures of different geographical distances/distance indices, expressed as isolation by author
Fragmentation			relative fragmentation, indices, edge density	27	Measures that describe degree of fragmentation according to authors, including both landscape and patch measures
Shape			indices, edge/area ratio, patch shape, edge density	11	Measures that describe habitat shape in combination with authors expression
Configuration			PC-measures, distributions, aggregation, landscape structure, indices	23	Measures that spatially describe the configuration of or relation between different habitat types in a landscape context
Quality			proportional cover/openness, indices, heterogeneity	27	Relevant measures of habitat structures in relation to studied organism
**Ecological processes**					
Demographic	*Reproduction*	+	nesting/fledgling success, seed germination, birth rate, number of seeds	292	Various measures of reproductive success relevant for the organism
Demographic	*Population growth*	+	growth rate, trend, recovery rate	19	Measures of population growth without specified mechanism
Persistence	*Survival*	+	survival rate, years of survival, condition, winter/summer survival	21	Measures of adult survival
Persistence	*Persistence*	+	population size, population density, occupation probability, turnover	16	Population level persistence as measured by authors
Persistence	*Extinction*	-	probability/rate/risk/time of extinction	15	Measures of estimations of population level extinction events
Persistence	*Mortality*	-	time until mortality, mortality risk/probability/rate, pathogen occurrence	28	Individual level mortality measures with population level effects
Dispersal	*Dispersal*	+	genetic measures, dispersal distances, dispersal rate	160	Measures specified by author to be related to dispersal
Dispersal	*Colonization*	+	colonization rate/probability/speed/success	26	Movement measures specified by authors as colonization events
Dispersal	*Emigration*		emigration rate/propensity/probability/decision	10	Movement measures specified by authors as emigration events
Dispersal	*Immigration*	+	recruitment rate, number of immigrations, immigration probability	24	Movement measures specified by authors as immigration events

Thereafter, we classified observed relationships between habitat attributes and population viability as “positive”, “negative”, “non-significant effect”, or as “inconclusive”, based on results reported in articles. Quality aspects, e.g., canopy cover, habitat structure, habitat heterogeneity index etc. (see Table S3), did not match our original terms in the search string, but since quality effects were identified during the systematic process and are of interest for the general discussion on population persistence, these results were extracted and included in the analysis. Illustrations were produced for a summary of the data, showing both the number of unique articles and the number of specific cases from the identified articles, and for subgroups based on taxonomic group.

Results

The results are summarized and presented as rounded up percentage of total number of cases found relevant for each identified category of variables that we have specified for habitat attributes, ecological processes and for different taxonomic groups. The result sections below first address general patterns of how studies fall within and between different categories. The second section of the results address the direction of effects we have found on population viability between habitat attributes and ecological processes. We present the most evident patterns in the result section, results based on few cases are only reported if found relevant for the aim of this study – to describe the distribution of underlying scientific evidence guiding practical conservation measures.

General patterns: Habitat attributes, ecological processes, and taxonomy

The most studied habitat attribute category in the data set was patch area (34%), followed by isolation (18%), fragmentation (16%), habitat amount (12%), connectivity (8%), with fewer cases for other attributes (see Figure S3). Quality aspects were found in 5% of all cases. The overall most studied ecological process was reproduction (46%), followed by dispersal (22%), colonization (9%), and extinction (6%) (Figure S3). Fewer cases were represented for other ecological processes. Summarized on the three main categories for ecological processes, demographic effects were studied in 49% of the cases, dispersal effects in 34% of the cases, and population persistence effects in 17% of the cases. The two dominating taxonomic groups were plants and birds (38% and 34% respectively), followed by insects (14%) and mammals (10%). A few cases were identified for amphibians and reptiles, molluscs, spiders, and fungi (Figure S3).

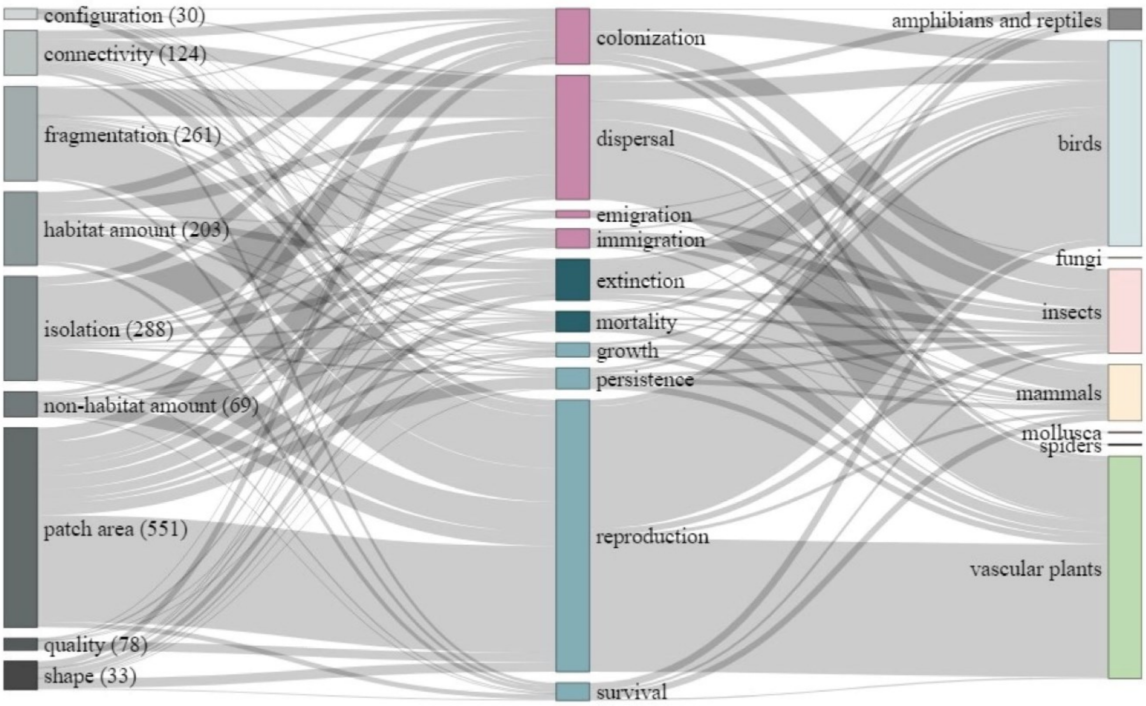

Figure S3. Distribution of proportions of identified cases (in total 1637 cases from 342 unique studies) and studied relationships between habitat attributes, ecological processes, and taxonomic groups.

The number of cases of studied relationships between habitat attributes and ecological processes varied. The most well studied relationship was that between patch area and reproduction (18%), followed by fragmentation and reproduction (9%), isolation and dispersal (8%), habitat amount and reproduction (6%), fragmentation and dispersal (6%) (Figure S3). Other relationships were studied to a lesser extent.

The two dominating taxonomic groups vascular plants and birds explained the overall patterns described above. The patterns for the less dominating taxonomic groups of insects and mammals differed slightly from that of vascular plants and birds, such that they were studied relatively more in relation to movement processes (dispersal, immigration, emigration, and colonization) rather than demographic processes (population growth and reproduction) (65 % and 60 % respectively of all cases for insects and mammals).

Direction of effects: habitat attributes and ecological processes

The overall pattern of *patch area* effects on population viability related processes demonstrated a majority of results with no non-significant effects (53% of the cases) and positive effects (31% of the cases) being more common than negative effects (10% of the

cases) while inconclusive records were relatively few. Patch area was most studied in relation to reproduction with non-significant effects in most cases (60% of the cases), followed by positive effects with increasing patch area (25% of the cases). Fewer cases showed negative (8% of the cases) or inconclusive effects (7% of the cases). Other demographic processes were represented by relatively few cases (see Figure S4a). Patch area related to movement processes constituted 24% of the cases. Here, most of the identified cases related to dispersal, with most of the results showing no effect of increasing patch area (57% of the cases), followed by positive dispersal effects (21% of the cases) and fewer with negative dispersal effects (14% of the cases). Other movement processes were studied less frequently.

Habitat amount also showed most results with non-significant effects (48% of the cases), followed by positive effects (42% of the cases), negative effects (7% of the cases) and only a few inconclusive cases (Figure S4b). Habitat amount was mainly studied in relation to demographic processes (47% of the cases), with a dominance of effects on reproduction (46% of the cases). Reproduction showed non-significant effects in 57% of the cases, positive effects in 37% of the cases and negative or inconclusive effects in 3% of the cases respectively. Most other studied ecological processes followed the same pattern as reproduction, with a slight dominance of results with non-significant effect, followed by increasing effects, and some cases with decreasing effects. Dispersal, however, was instead dominated by positive effects with increasing habitat amount (67% of the cases).

For the cases related to *quality* aspects, most studies showed non-significant effects (53% of the cases), followed by positive effects (38% of the cases) and negative effects (6% of the cases) (Figure S4c). Quality was mostly studied in relation to demographic processes and in particular reproduction, with positive effects in 41% of the cases and negative in 7% of the cases. Movement processes, dominated by colonization, was the second most studied process type with non-significant effects on colonization in 59% of the cases, positive effects in 29% of the cases, and negative or inconclusive effects in 6% of the cases. Persistence processes, dominated by extinction, were almost as well represented as movement processes, showing non-significant effects on extinction in 67% of the cases, positive effects in 27% of the cases, 7% of the cases were inconclusive, and no cases with negative effects.

Connectivity showed similar proportions of results with positive effects (46% of the cases) and non-significant effects (40% of the cases) on population viability determinants, followed by negative effects (11% of the cases), and a few cases with inconclusive results (Figure

S4d). Dispersal was the most studied process in relation to connectivity (31% of the cases), followed by reproduction (23%) and colonization (20%). Most of the cases showed positive effects on dispersal related response variables (55% of the cases), while fewer cases showed non-significant effect (34% of the cases) or negative effects (11%) on dispersal (Figure S4d). Colonization also showed mostly positive effects from connectivity (44%), followed by non-significant effect (32%), and negative effects (24%), while reproduction effects were more difficult to conclude (54% of the cases showing non-significant effect, 25% showing positive reproductive effects, and 14% showing negative effects).

Isolation showed mostly non-significant effects on ecological processes (53% of the cases), followed by negative effects (34% of the cases), positive effects (10% of the cases) and few cases with inconclusive results. Isolation was most well studied in relation to movement processes (56 % of the cases), with dispersal being the dominant process (Figure S4e). Isolation showed a negative effect on dispersal in most cases (53% of the cases), followed by no effect (44% of the cases), and with few inconclusive or positive effects. Other movement processes were studied in relatively few cases. Reproduction was the most well studied demographic process in relation to isolation (29% of the cases), with most results showing non-significant effect (56% of the cases), and similar proportions of positive and negative effects (19 and 18 % of the cases respectively). Other demographic processes were less well represented in relation to isolation.

Fragmentation showed a majority of results with non-significant effects (50% of the cases), followed by negative effects (34% of the cases), and positive effects (10% of the cases) (Figure S4f). Reproduction was the most studied process in relation to fragmentation (55% of the cases), followed by effects on dispersal (29% of the cases). Reproduction most often showed non-significant effects from fragmentation (53% of the cases), followed by a negative effect (33%), and in 9% of the cases fragmentation showed a positive reproductive effect. Similar patterns could be seen for dispersal with 49% of the cases showing non-significant effects, 36 % of the cases showing negative effects, and 7% of the cases showing positive dispersal effects from fragmentation while 9% of the cases were inconclusive. Other ecological processes were less well represented in the results.

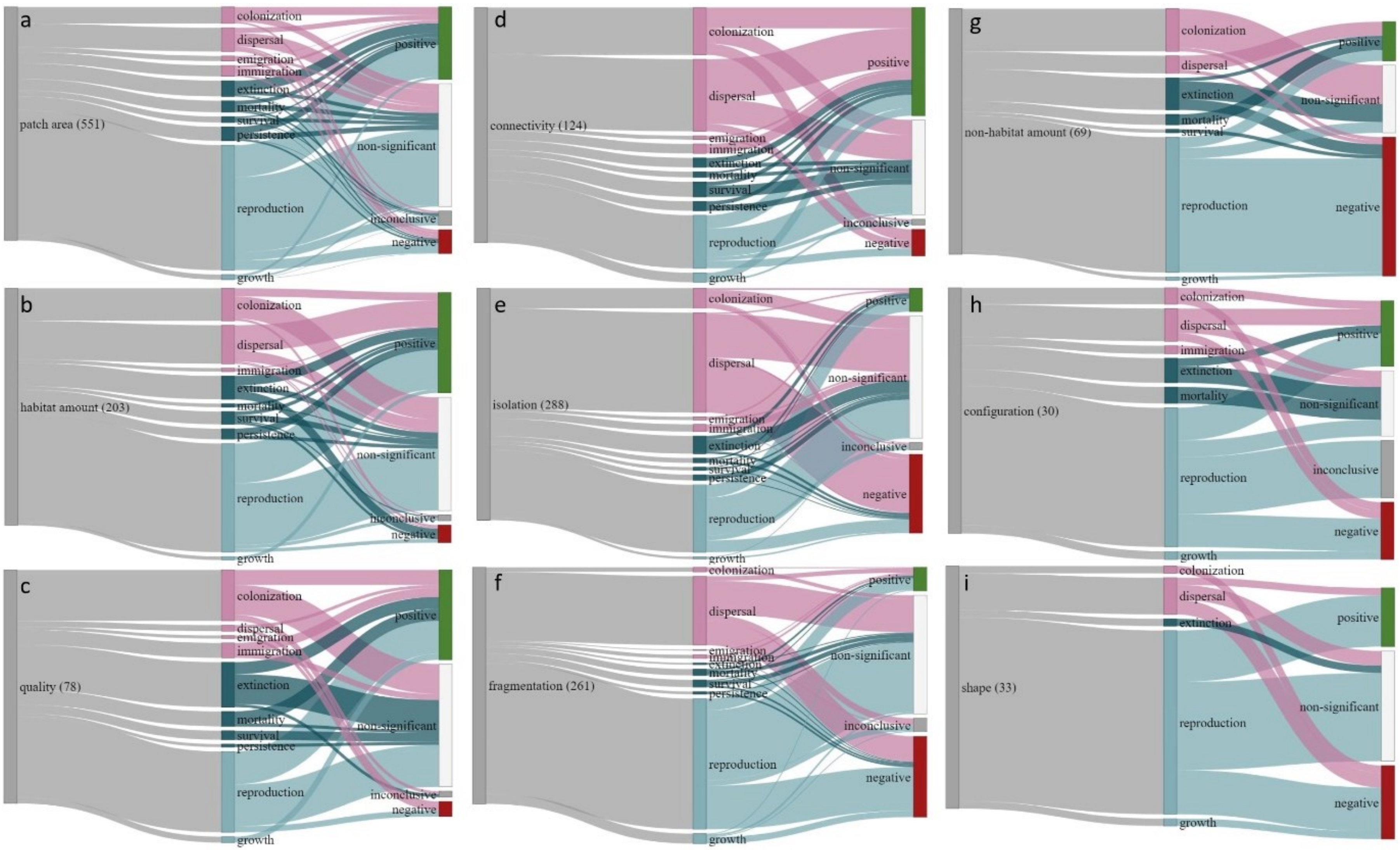

Figure S4. Habitat attributes studied in relation to different ecological processes and their effects on population viability. The effects on extinction and mortality were reversely included to correspond to the net effect on population viability. Figure showing proportions of results from the total number of result cases identified. Ecological processes are classified in three different types, represented with separate colours; movement processes in pink, population persistence in dark turquoise, and productivity processes are shown in light turquoise.

Most of the *non-habitat amount* cases showed negative effects on ecological processes (57% of the cases), followed by non-significant effects (28% of the cases), and positive effects (16% of the cases) (Figure S4g). Non-habitat amount was for the most part studied in relation to demographic or persistence processes (75% of the cases vs. 25% of the cases were movement related) (Figure S4h). Demographic studies were dominated by effects on reproduction (55%). Most of the reproductive results showed negative effects (84% of the cases), few cases showed positive or non-significant effects (8% of cases respectively) on reproduction. Colonization was the most studied movement process (17% of the cases), with 92% of the cases showing non-significant effects and one case showing a negative effect.

Configuration and shape were the least studied habitat attributes, with shape dominated by non-significant effect results on processes (45% of the cases), followed by similar amounts of results with positive (24% of the cases) or negative effects (30% of the cases), and configuration showing equal distributions between positive, non-significant effect, inconclusive and negative effects (24-27% of the cases) (Figure S4h and S4i). For both types of habitat attributes, reproduction was the most studied ecological process (shape 76% of the cases, and configuration 57% of the cases). Shape showed mixed effects on reproduction; 48% of the cases showing non-significant effects on reproduction, 28% of the cases positive effects, and 24% negative effects. Configuration effects were inconclusive in 41% of the cases, had either positive or negative effect on reproduction in 24% of the cases respectively, and 12% of the cases showed non-significant effects. However, the number of cases were generally low (2-17 cases in each direction-effect category). Dispersal was the second most studied process in relation to both shape and configuration (15% and 13% of the cases for shape and configuration respectively), showing similar mixed results as reproduction effects although based on very few cases (4-5 cases). Other processes e.g., colonization, immigration, extinction, mortality, and growth were only represented with very few cases each and are therefore not summarized further.

References

Allaire, J.J, C. Gandrud, K. Russell, and C.J. Yetman. 2017. networkD3: D3 JavaScript Network Graphs from R. R package version 0.4. https://CRAN.R-project.org/package=networkD3

Ekroos, J., von Post, M., Nilsson, L., and H.G. Smith. 2020. Effects of green infrastructure on biodiversity – a review. Swedish Environmental Protection Agency, Report 6922, Stockholm Sweden (in Swedish, English summary) (Report).

Luederitz, C., Meyer, M., Abson, D.J., Gralla, F., Lang, D.J., Rau, A.L. and H. von Wehrden. 2016. Systematic student-driven literature reviews in sustainability science–an effective way to merge research and teaching. Journal of Cleaner Production, 119, pp.229-235.

R Core Team (2021). R: A language and environment for statistical computing. R Foundation for Statistical Computing, Vienna, Austria.URL https://www.R-project.org/.

Appendix S1. Metadata collected for scientific publications on GI and biodiversity

MAIN CATEGORIES	DATA NOTED	EXPLANAITION
**ID**	ID for this study	From endnote
**AUTHOR**	Authors	From endnote
**YEAR**	Year published	From endnote
**TITLE**	Title	From endnote
**JOURNAL**	Name of journal	From endnote
**VOLUME;NO**	Volume and number of journal	From endnote
**PAGES**	Pages	From endnote
**ABSTRACT**	Abstract	From endnote
**GI CONTEXT (1/0)**	1/0	1 = GI mentioned in abstract 0=GI not included in abstract
**RELEVANT AS BACKGROUND BASED ON TITLE AND ABSTRACT (1/0)**	1/0	GI focus dominant in abstract regardless of location
**LOCATION**	Country and continent	
**CLIMATE ZONE**	Cimate zone; tempered;tropical;subtropical;arctic etc.	Only studies from temperate zones are included
**MAIN FOCUS**	Main objective with GI from authors perspective; Biodiversity/Climate adaptation/Climate regulation/Recreation/Green economy/Multifunctionality/Ecosystem services unspecified	Only studies including aspects of biodiversity or biodiversity related ecosystem services are included
**INCLUDE**	Yes/no/maybe	If yes or maybe --> fulltext
**REASON FOR EXCLUSION**	Location/Climate zone/Main focus	Name the missing criteria(s) (Location/Climate zone/Biodiversity aspects)
**BIODIVERSITY CONTEXT**	Is biodiversity quantified, 1/0	Is there a quantitative measure of biodiversity, regardless of measured unit
	Biodiversity reference (biodiversity, species composition, functional group, taxonomic group, area etc.)	What is the biodiversity reference/measure in the study
	Taxonomic group or habitat type	Ex. mammals, insects, birds, forests, grasslands, plants
**PUBLICATION TYPE**	Review, original article	
	Empirical, opinion, theoretical, secondary data	If original, is it an empirical, theoretical, opinion paper or metanalysis etc.
**GI CONTEXT**	Abstract/Physical	If GI referred to as abstract (strategy) or physical structure

Table A1. Metadata collected for scientific publications on GI and biodiversity

	If physical, describe what type of GI	Ex. wetland, ecoduct, green roof, forest, raingarden, grassland etc.
**MAIN LANDUSE**	Main land use according to LUCAS	Use LUCAS landcover; artificial land;cropland; woodland;shrubland; grassland;bare land and lichens/moss;inland water;coastal water.
	Main land use as described by authors	
**PREFERRED HABITAT**	The organism's natural habitat according to LUCAS	Describing environmental type preferred by the study organism. Use LUCAS landcover; artificial land;cropland; woodland;shrubland; grassland;bare land and lichens/moss;inland water;coastal water
	The organism's natural habitat as described by author	Authors description of preferred habitat
**INCLUDES RECOMMENDATIONS RELATED TO BBMJ**	1/0	Can the conclusions drawn by authors be related to any of the following conservation strategies; Bigger areas, Better quality habitats, More habitats in general, better connected/Joined
	Better;Bigger;More;Joined	If 1 in previous, add term.

Appendix S2. Included publications on green infrastructure and biodiversity

Author	Year	Journal	Volume (Issue)	Title
**Albert, C. H. R., B.: Dumitru, M.: Gonzalez, A.**	2017	Conservation Biology	31(6)	"Applying network theory to prioritize multispecies habitat networks that are robust to climate and land-use change."
**Aloisio, J. M. P., M. I.: Giampieri, M. A.: Tuininga, A. R.: Lewis, J. D.**	2017	Ecological Applications	27(1)	"Spatially dependent biotic and abiotic factors drive survivorship and physical structure of green roof vegetation."
**Andersson, E. B., S.: Borgström, S.: Colding, J.: Elmqvist, T.: Folke, C.: Gren, A.**	2014	Ambio	43(4)	"Reconnecting cities to the biosphere: Stewardship of green infrastructure and urban ecosystem services."
**Andersson, E. C., J.**	2014	Urban Forestry & Urban Greening	13(2)	"Understanding how built urban form influences biodiversity."
**Angelstam, P. A., K.: Axelsson, R.: Elbakidze, M.: Jonsson, B. G.: Roberge, J. M.**	2011	Silva Fennica	45(5)	"Protecting Forest Areas for Biodiversity in Sweden 1991-2010: the Policy Implementation Process and Outcomes on the Ground."
**Angelstam, P. K., O.: Yamelynets, T.: Mozgeris, G.: Naumov, V.: Chmielewski, T. J.: Elbakidze, M.: Manton, M.: Prots, B.: Valasiuk, S.**	2017	Journal of Environmental Management	193	"Green infrastructure development at European Union's eastern border: Effects of road infrastructure and forest habitat loss."
**Angelstam, P. L., M.**	2017	Ecological Engineering	103	"Tall herb sites as a guide for planning, maintenance and engineering of riparian continuous forest cover."
**Angelstam, P. M., G.: Rönnbäck, B. I.: Östman, A.: Lazdinis, M.: Roberge, J. M.: Arnberg, W.: Olsson, J.**	2003	Ambio	32(8)	"Two-dimensional Gap Analysis: A Tool for Efficient Conservation Planning and Biodiversity Policy Implementation."
**Angelstam, P. M., M.: Khaulyak, O.: Naumov, V.: Pedersen, S.: Stryamets, N.: Tornblom, J.: Valasiuk, S.: Yamelynets, T.**	2019	Lesnoy Zhurnal-Forestry Journal	1	"KNOWLEDGE PRODUCTION AND LEARNING FOR SUSTAINABLE FOREST LANDSCAPES: THE EUROPEAN CONTINENT'S WEST AND EAST AS A LABORATORY."
**Angelstam, P. N., V.: Elbakidze, M.: Manton, M.: Priednieks, J.: Rendenieks, Z.**	2018	Ecosphere	93	"Wood production and biodiversity conservation are rival forestry objectives in Europe's Baltic Sea Region."
**Angelstam, P. P., S.: Manton, M.**	2018	Lesnoy Zhurnal-Forestry Journal	4	"MACROECOLOGICAL RESEARCH IN BOREAL FOREST REVEALS THE EFFECTS OF MOOSE ON ECONOMICALLY AND ECOLOGICALLY IMPORTANT TREE SPECIES."
**Angelstam, P. P., S.: Manton, M.: Garrido, P.: Naumov, V.: Elbakidze, M.**	2017	Landscape and Urban Planning	167	"Green infrastructure maintenance is more than land cover: Large herbivores limit recruitment of key-stone tree species in Sweden."
**Angelstam, P. Y., T.: Elbakidze, M.: Prots, B.: Manton, M.**	2017	Ecoscience	24(1-2)	"Gap analysis as a basis for strategic spatial planning of green infrastructure: a case study in the Ukrainian Carpathians."
**Artmann, M. B., O.: Grunewald, K.**	2017	Sustainability	9(2)	"Using the Concepts of Green Infrastructure and Ecosystem Services to Specify Leitbilder for Compact and Green Cities-The Example of the Landscape Plan of Dresden (Germany)."
**Artmann, M. S., K.**	2018	Sustainability	10(6)	"The Role of Urban Agriculture as a Nature-Based Solution: A Review for Developing a Systemic Assessment Framework."
**Balbi, M. P., E. J.: Croci, S.: Nabucet, J.: Georges, R.: Madec, L.: Ernoult, A.**	2019	Journal of Environmental Management	244	"Title: Ecological relevance of least cost path analysis: An easy implementation method for landscape urban planning."
**Bell, G. N., S.: Medcalf, K.**	2015	Ecological Informatics	30	"Use of remote sensing to produce a habitat map of Norfolk."

Appendix S2. Included publications on green infrastructure and biodiversity

**Bellamy, C. C. v. d. J., A. P. N.: Barbour, S.: Smith, M.: Moseley, D.**	2017	Environmental Research	158	"A spatial framework for targeting urban planning for pollinators and people with local stakeholders: A route to healthy, blossoming communities?"
**Beumer, C.**	2018	Urban Forestry & Urban Greening	30	"Show me your garden and I will tell you how sustainable you are: Dutch citizens' perspectives on conserving biodiversity and promoting a sustainable urban living environment through domestic gardening."
**Beumer, C. M., P.**	2015	Sustainability Science	10(1)	"Biodiversity in my (back)yard: towards a framework for citizen engagement in exploring biodiversity and ecosystem services in residential gardens."
**Biffi, S. D. S., C. M.: Firbank, L. G.**	2019	Agriculture, Ecosystems and Environment	286	"Epigeal fauna of urban food production sites show no obvious relationships with soil characteristics or site area."
**Blanusa, T. G., M.: Cathcart-James, M.: Hunt, L.: Cameron, R. W. F.**	2019	Urban Forestry & Urban Greening	44	"Urban hedges: A review of plant species and cultivars for ecosystem service delivery in north-west Europe."
**Blasi, C. C., G.: Orti, M. M. A.: Anzellotti, I.: Attorre, F.: Azzella, M. M.: Carli, E.: Copiz, R.: Garfi, V.: Manes, F.: Marando, F.: Marchetti, M.: Mollo, B.: Zavattero, L.**	2017	Environmental Science & Policy	78	"Ecosystem mapping for the implementation of the European Biodiversity Strategy at the national level: The case of Italy."
**Boc, V. I.**	2015	Scientific Papers-Series B-Horticulture	59	"CURRENT APPROACHES IN METROPOLITAN GREEN INFRASTRUCTURE STRATEGIES."
**Bormpoudakis, D. T., J.**	2019	Landscape Ecology	34(11)	"The science-practice interface of connectivity in England."
**Borysiak, J. M., A.: Speak, A.**	2017	Urban Ecosystems	20(2)	"Floral biodiversity of allotment gardens and its contribution to urban green infrastructure."
**Botzat, A. F., L. K.: Kowarik, I.**	2016	Global Environmental Change-Human and Policy Dimensions	39	"Unexploited opportunities in understanding liveable and biodiverse cities. A review on urban biodiversity perception and valuation."
**Brunbjerg, A. K. H., J. D.: Bates, A. J.: Fowler, R. E.: Rosenfeld, E. J.: Sadler, J. P.**	2018	Urban Forestry & Urban Greening	32	"Can patterns of urban biodiversity be predicted using simple measures of green infrastructure?"
**Bugnot, A. B. M.-P., M.: Johnston, E. L.: Schaefer, N.: Dafforn, K. A.**	2018	Ecological Engineering	120	"Learning from nature to enhance Blue engineering of marine infrastructure."
**Buijs, A. E. M., T. J.: Van der Jagt, A. P.: Ambrose-Oji, B.: Andersson, E.: Elands, B. H.: Steen Møller, M.**	2016	Current Opinion in Environmental Sustainability	22	"Active citizenship for urban green infrastructure: fostering the diversity and dynamics of citizen contributions through mosaic governance."
**Butt, N. S., D. F.: Shumway, N.: Bekessy, S. A.: Fuller, R. A.: Watson, J. E. M.: Maggini, R.: Hole, D. G.**	2018	Geo-Geography and Environment	5(1)	"Opportunities for biodiversity conservation as cities adapt to climate change."
**Capotorti, G. A. O., M. M.: Anzellotti, I.: Azzella, M. M.: Copiz, R.: Mollo, B.: Zavattero, L.**	2015	Plant Biosystems	149(6)	"The MAES process in Italy: Contribution of vegetation science to implementation of European Biodiversity Strategy to 2020."
**Carlier, J. M., J.**	2019	Science of the Total Environment	651	"Landscape typology and ecological connectivity assessment to inform Greenway design."
**Carlier, J. M., J.: Aughney, T.: Roche, N.**	2019	Global Ecology and Conservation	18	"Effects of greenway development on functional connectivity for bats."
**Casalegno, S. A., K.: Hancock, S.: Gaston, K. J.**	2017	Methods in Ecology and Evolution	8(11)	"Improving models of urban greenspace: from vegetation surface cover to volumetric survey, using waveform laser scanning."

Appendix S2. Included publications on green infrastructure and biodiversity

**Catalano, C. L., V. A.: Badalucco, L.: Guarino, R.**	2018	Ecological Engineering	115	"Some European green roof norms and guidelines through the lens of biodiversity: Do ecoregions and plant traits also matter?"
**Closset-Kopp, D. W., S.: Decocq, G.**	2016	Biological Conservation	201	"Using process-based indicator species to evaluate ecological corridors in fragmented landscapes."
**Collins, R. S., M.: Hudson, M. D.**	2017	Land Use Policy	64	"The value of green walls to urban biodiversity."
**Connery, K.**	2009	Journal of Green Building	4(2)	"Biodiversity and urban design: Seeking an integrated solution."
**Connop, S. V., P.: Eisenberg, B.: Collier, M. J.: Nash, C.: Clough, J.: Newport, D.**	2016	Environmental Science & Policy	62	"Renaturing cities using a regionally-focused biodiversity-led multifunctional benefits approach to urban green infrastructure."
**Cox, D. T. C. B., J.: Casalegno, S.: Hudson, H. L.: Anderson, K.: Gaston, K. J.**	2019	Landscape and Urban Planning	185	"Skewed contributions of individual trees to indirect nature experiences."
**Cox, D. T. C. H., H. L.: Plummer, K. E.: Siriwardena, G. M.: Anderson, K.: Hancock, S.: Devine-Wright, P.: Gaston, K. J.**	2018	Journal of Applied Ecology	55(5)	"Covariation in urban birds providing cultural services or disservices and people."
**Dallimer, M. D., Z. G.: Diaz-Porras, D. F.: Irvine, K. N.: Maltby, L.: Warren, P. H.: Armsworth, P. R.: Gaston, K. J.**	2015	Global Environmental Change-Human and Policy Dimensions	31	"Historical influences on the current provision of multiple ecosystem services."
**Dallimer, M. T., Z. Y.: Gaston, K. J.: Davies, Z. G.**	2016	Ecology and Evolution	6(7)	"The extent of shifts in vegetation phenology between rural and urban areas within a human-dominated region."
**Davies, Z. G. F., R. A.: Loram, A.: Irvine, K. N.: Sims, V.: Gaston, K. J.**	2009	Biological Conservation	142(4)	"A national scale inventory of resource provision for biodiversity within domestic gardens."
**Davis, A. Y. B., J. A.: Farfan, M. A.: Milz, D.: Sweeney, E. R.: Loss, S. R.: Minor, E. S.**	2012	Ecosphere	3(11)	"Green infrastructure and bird diversity across an urban socioeconomic gradient."
**Dawson, L. E., M.: Angelstam, P.: Gordon, J.**	2017	Journal of Environmental Management	197	"Governance and management dynamics of landscape restoration at multiple scales: Learning from successful environmental managers in Sweden."
**de la Fuente, B. M.-S., M. C.: Rodriguez, G.: Gaston, A.: de Ayala, R. P.: Colomina-Perez, D.: Melero, M.: Saura, S.**	2018	Land Use Policy	75	"Natura 2000 sites, public forests and riparian corridors: The connectivity backbone of forest green infrastructure."
**Deslauriers, M. R. A., A.: Nazarnia, N.: Jaeger, J. A. G.**	2018	Ecological Indicators	94	"Implementing the connectivity of natural areas in cities as an indicator in the City Biodiversity Index (CBI)."
**Diduck, A. P. R., C. M.: Rodela, R.: Moquin, R.: Boerchers, M.**	na	Journal of Environmental Planning and Management		"Pathways of learning about biodiversity and sustainability in private urban gardens"
**Do, Y. L., M.: Joo, G. J.**	2014	Urban Ecosystems	17(3)	"Carabid beetles in green infrastructures: the importance of management practices for improving the biodiversity in a metropolitan city."
**Donaldson, G. H. J., E. M.**	2019	Impact Assessment and Project Appraisal		"Using green infrastructure to add value and assist place-making in public realm developments."
**Dreher, D.**	2009	Journal of Green Building	4(3)	"CHICAGO WILDERNESS GREEN INFRASTRUCTURE VISION: CHALLENGES AND OPPORTUNITIES FOR THE BUILT ENVIRONMENT."
**Dupras, J. D., C.: Andre, P.: Gonzalez, A.**	2015	Planning Practice and Research	30(4)	"Towards the Establishment of a Green Infrastructure in the Region of Montreal (Quebec, Canada)."

Appendix S2. Included publications on green infrastructure and biodiversity

**Dylewski, Ł. M., Ł: Banaszak-Cibicka, W.**	2019	Ecological Entomology	44(5)	"Are all urban green spaces a favourable habitat for pollinator communities? Bees, butterflies and hoverflies in different urban green areas."
**Elbakidze, M. A., P.: Yamelynets, T.: Dawson, L.: Gebrehiwot, M.: Stryamets, N.: Johansson, K. E.: Garrido, P.: Naumov, V.: Manton, M.**	2017	Landscape and Urban Planning	168	"A bottom-up approach to map land covers as potential green infrastructure hubs for human well-being in rural settings: A case study from Sweden."
**Elbakidze, M. R., R.: Manton, M.: Angelstam, P.: Mozgeris, G.: Brūmelis, G.: Brazaitis, G.: Vogt, P.**	2016	European Journal of Forest Research	135(2)	"The role of forest certification for biodiversity conservation: Lithuania as a case study."
**Elek, Z. H., A. G.: Enggaard, M. K.: Lövei, G. L.**	2017	Entomologica Fennica	28(1)	"Seasonal dynamics of common ground beetles (Coleoptera: Carabidae) along an urbanisation gradient near Sorø, Zealand, Denmark."
**Fairbrass, A. J. R., P.: Williams, C.: Titheridge, H.: Jones, K. E.**	2017	Ecological Indicators	83	"Biases of acoustic indices measuring biodiversity in urban areas."
**Fenu, G. P., P. L.**	2016	2016 International Multidisciplinary Conference on Computer and Energy Science, SpliTech 2016,		A land similarity approach to modeling complex ecological networks.
**Filazzola, A. S., N.: MacIvor, J. S.**	2019	Journal of Applied Ecology	56(9)	"The contribution of constructed green infrastructure to urban biodiversity: A synthesis and meta-analysis."
**Fischer, L. K. B., D.: Karle, S. J.: Cremer, K.: Huttner, E.: Seebauer, M.: Nowikow, U.: Schütze, B.: Voigt, P.: Völker, S.: Kowarik, I.**	2019	Urban Forestry and Urban Greening	40	"Biodiverse edible schools: Linking healthy food, school gardens and local urban biodiversity."
**Fischer, L. K. E., J.: Kowarik, I.: Buchholz, S.**	2016	Peerj	4	"Disentangling urban habitat and matrix effects on wild bee species."
**Fischer, L. K. v. d. L., M.: Kowarik, I.**	2013	Urban Forestry & Urban Greening	12(3)	"Urban land use types contribute to grassland conservation: The example of Berlin."
**Fulthorpe, R. M., J. S.: Jia, P.: Yasui, S. L. E.**	2018	Frontiers in Ecology and Evolution	6	"The Green Roof Microbiome: Improving Plant Survival for Ecosystem Service Delivery."
**Fumagalli, N. T., A.**	2012	International Journal of Environmental Research	6(4)	"Relationship between greenways and ecological network: A case study in Italy."
**Funk, A. M.-L., J.: Borgwardt, F.: Trauner, D.: Bagstad, K. J.: Balbi, S.: Magrach, A.: Villa, F.: Hein, T.**	2019	Science of the Total Environment	654	"Identification of conservation and restoration priority areas in the Danube River based on the multi-functionality of river-floodplain systems."
**Garcia-Feced, C. W., C. J.: Baraldi, A.: Paracchini, M. L.: Maes, J.: Zulian, G.: Kempen, M.: Elbersen, B.: Perez-Soba, M.**	2015	Agronomy for Sustainable Development	35(1)	"Semi-natural vegetation in agricultural land: European map and links to ecosystem service supply."
**Garmendia, E. A., E.: Adams, W. M.: Bormpoudakis, D.**	2016	Land Use Policy	56	"Biodiversity and Green Infrastructure in Europe: Boundary object or ecological trap?"
**Gavrilidis, A. A. N., M. R.: Onose, D. A.: Badiu, D. L.: Năstase, I. I.**	2019	Ecological Indicators	96	"Methodological framework for urban sprawl control through sustainable planning of urban green infrastructure."
**Gill, A. S. L., A.: McGuire, K. L.**	2017	Applied and Environmental Microbiology	83(16)	"Phylogenetic and Functional Diversity of Total (DNA) and Expressed (RNA) Bacterial Communities in Urban Green Infrastructure Bioswale Soils."
**Giupponi, L. B., G.: Giorgi, A.: Bischetti, G. B.**	2019	Landscape and Ecological Engineering	15(1)	"How to renew soil bioengineering for slope stabilization: some proposals."

Appendix S2. Included publications on green infrastructure and biodiversity

**Grashof-Bokdam, C. J. C., A.: Polman, N. B. P.: Westerhof, Ejgm: Franke, J. G. J.: Opdam, P. F. M.**	2017	Landscape Ecology	32(3)	"Modelling shifts between mono- and multifunctional farming systems: the importance of social and economic drivers."
**Green, O. O. G., A. S.: Albro, S.: Ban, N. C.: Berland, A.: Burkman, C. E.: Gardiner, M. M.: Gunderson, L.: Hopton, M. E.: Schoon, M. L.: Shuster, W. D.**	2016	Urban Ecosystems	19(1)	"Adaptive governance to promote ecosystem services in urban green spaces."
**Green, T. L. K., J.: Andersson, E.: Elmqvist, T.: Gomez-Baggethun, E.**	2016	Ecosystems	19(6)	"Insurance Value of Green Infrastructure in and Around Cities."
**Grunwald, L. H., J.: Weber, S.**	2017	Urban Forestry and Urban Greening	22	"A GIS-based mapping methodology of urban green roof ecosystem services applied to a Central European city."
**Haaland, C.**	2017	Journal of Insect Conservation	21(5-6)	"How to preserve a butterfly species within an urbanising settlement and its surroundings: a study of the scarce copper (Lycaena virgaureae L.) in southern Sweden."
**Hale, J. D. S., J.**	2012	Proceedings of the Institution of Civil Engineers-Engineering Sustainability	165(1)	"Resilient ecological solutions for urban regeneration."
**Hatziiordanou, L. F., E.: Hadjicharalampous, E.: Eleftheria Votsi, N.: Palaskas, D.: Abdul Malak, D.**	2019	One Ecosystem	4	"Indicators for mapping and assessment of ecosystem condition and of the ecosystem service habitat maintenance in support of the EU biodiversity strategy to 2020."
**Hauck, J. S., J.: Werner, A.**	2016	Ecology and Society	21(2)	"Using social network analysis to identify key stakeholders in agricultural biodiversity governance and related land-use decisions at regional and local level."
**Hodge, I. H., J.: Bonn, A.**	2015	Conservation Biology	29(4)	"The alignment of agricultural and nature conservation policies in the European Union."
**Hofmann, M. W., J. R.: Kowarik, I.: van der Meer, E.**	2012	Urban Forestry & Urban Greening	11(3)	"Perceptions of parks and urban derelict land by landscape planners and residents."
**Holt, A. R. M., M.: Maltby, L.: Warren, P.**	2015	Ecosystem Services	16	"Understanding spatial patterns in the production of multiple urban ecosystem services."
**Horák, J. R., J.: Rada, P.: Šafářová, L.: Koudelková, J.: Zasadil, P.: Halda, J. P.: Holuša, J.**	2018	Urban Ecosystems	21(2)	"Renaissance of a rural artifact in a city with a million people: biodiversity responses to an agro-forestry restoration in a large urban traditional fruit orchard."
**Hornigold, K. L., I.: Dolman, P.**	2016	Plos One	11(11)	"Recreational Use of the Countryside: No Evidence that High Nature Value Enhances a Key Ecosystem Service."
**Hostetler, M. A., W.: Meurk, C.**	2011	Landscape and Urban Planning	100(4)	"Conserving urban biodiversity? Creating green infrastructure is only the first step."
**Hoyle, H. H., J.: Jorgensen, A.**	2017	Landscape and Urban Planning	164	"All about the 'wow factor'? The relationships between aesthetics, restorative effect and perceived biodiversity in designed urban planting."
**Hoyle, H. J., A.: Warren, P.: Dunnett, N.: Evans, K.**	2017	Urban Forestry & Urban Greening	25	""Not in their front yard" The opportunities and challenges of introducing perennial urban meadows: A local authority stakeholder perspective."
**Hoyle, H. N., B.: Dunnett, N.: Richards, J. P.: Russell, J. M.: Warren, P.**	2018	Landscape and Urban Planning	180	"Plant species or flower colour diversity? Identifying the drivers of public and invertebrate response to designed annual meadows."
**Hunter, M. C. R. B., D. G.**	2012	Landscape and Urban Planning	105(4)	"Spatial contagion: Gardening along the street in residential neighborhoods."

Appendix S2. Included publications on green infrastructure and biodiversity

**Ignatieva, M. A., K.**	2013	Journal of Architecture and Urbanism	37(1)	"Biodiverse green infrastructure for the 21st century: From "green desert" of lawns to biophilic cities."
**Jakobsson, S. F., K.: Cousins, S. A. O.**	2016	Journal of Vegetation Science	27(1)	"Connectivity and management enables fast recovery of plant diversity in new linear grassland elements."
**Jeusset, A. V., M.: Bertheau, Y.: Coulon, A.: Deniaud, N.: Flamerie De Lachapelle, F.: Jaslier, E.: Livoreil, B.: Roy, V.: Touroult, J.: Vanpeene, S.: Witté, I.: Sordello, R.**	2016	Environmental Evidence	5(1)	"Can linear transportation infrastructure verges constitute a habitat and/or a corridor for biodiversity in temperate landscapes? A systematic review protocol."
**Johansson, V. K., A.: Hedblom, M.: Deboni, G.: Andersson, P.**	2018	Ecosphere	9(10)	"Estimates of accessible food resources for pollinators in urban landscapes should take landscape friction into account."
**Jonsson, B. G. S., J.: Mikusiński, G.: Manton, M.: Angelstam, P.**	2019	Forests	10(7)	"European Union's last intact forest landscapes are at a value chain crossroad between multiple use and intensified wood production."
**Jorgensen, A. G., P. H.**	2010	Nature + Culture	5(3)	"Shades of Green: Measuring the Ecology of Urban Green Space in the Context of Human Health and Well-Being."
**Joyner, J. L. K., J.: Deeb, M.: Lozefski, G.: Prithiviraj, B.: Paltseva, A.: McLaughlin, J.: Groffman, P.: Cheng, Z.: Muth, T. R.**	2019	Frontiers in Microbiology	10(MAY)	"Green infrastructure design influences communities of urban soil bacteria."
**Klaus, V. H.**	2013	Restoration Ecology	21(6)	"Urban Grassland Restoration: A Neglected Opportunity for Biodiversity Conservation."
**Klimanova, O. A. K., E. Y.: Kurbakovskaya, A. V.**	2016	Geography and Natural Resources	37(2)	"Assessing the Geoecological Functions of the Green Infrastructure in Cities of Canada."
**Knapp, S. S., S.: Zehnsdorf, A.**	2019	Sustainability (Switzerland)	11(20)	"Biodiversity impact of green roofs and constructed wetlands as progressive eco-technologies in urban areas."
**Kovendi-Jako, A. H., M.: Csecserits, A.: Hulber, K.: Szitar, K.: Wrbka, T.: Torok, K.**	2019	Applied Vegetation Science	22(1)	"Three years of vegetation development worth 30 years of secondary succession in urban-industrial grassland restoration."
**Kowarik, I.**	2019	Landscape and Urban Planning	184	"The "Green Belt Berlin": Establishing a greenway where the Berlin Wall once stood by integrating ecological, social and cultural approaches."
**Kowarik, I. B., S.: von der Lippe, M.: Seitz, B.**	2016	Urban Forestry & Urban Greening	19	"Biodiversity functions of urban cemeteries: Evidence from one of the largest Jewish cemeteries in Europe."
**Krasny, M. E. R., A.: Tidball, K. G.: Elmqvist, T.**	2014	Ecosystem Services	7	"Civic ecology practices: Participatory approaches to generating and measuring ecosystem services in cities."
**Kremer, P. H., Z.: Haase, D.: McPhearson, T.: Frantzeskaki, N.: Andersson, E.: Kabisch, N.: Larondelle, N.: Rall, E. L.: Voigt, A.: Baró, F.: Bertram, C.: Gómez-Baggethun, E.: Hansen, R.: Kaczorowska, A.: Kain, J. H.: Kronenberg, J.: Langemeyer, J.: Pauleit, S.: Rehdanz, K.: Schewenius, M.: Van Ham, C.: Wurster, D.: Elmqvist, T.**	2016	Ecology and Society	21(2)	"Key insights for the future of urban ecosystem services research."
**Kucera, T. K., P.: Veseley, P.**	2015	Biodiversity and Conservation	24(13)	"Diverse vegetation in a spa town supports human social benefits of urban birds."
**Kukkala, A. S. M., A.**	2017	Landscape Ecology	32(1)	"Ecosystem services and connectivity in spatial conservation prioritization."
**Kuttner, M. H.-R., C.: Hermann, A.: Wrbka, T.**	2013	Ecological Indicators	31	"Borders without barriers - Structural functionality and green infrastructure in the Austrian-Hungarian transboundary region of Lake Neusiedl."

Appendix S2. Included publications on green infrastructure and biodiversity

**Kyrö, K. B., S.: Kotze, D. J.: Szallies, A.: Gerner, M.: Lehvävirta, S.**	2018	Urban Forestry and Urban Greening	29	"Local habitat characteristics have a stronger effect than the surrounding urban landscape on beetle communities on green roofs."
**Lahde, E. K., A.: Tahvonen, O.: Kokkonen, T.**	2019	Sustainability	11(7)	"Can We Really Have It All?Designing Multifunctionality with Sustainable Urban Drainage System Elements."
**Landor-Yamagata, J. L. K., I.: Fischer, L. K.**	2018	Sustainability	10(6)	"Urban Foraging in Berlin: People, Plants and Practices within the Metropolitan Green Infrastructure."
**Lennon, M. S., M.**	2014	Town Planning Review	85(5)	"Delivering ecosystems services via spatial planning: Reviewing the possibilities and implications of a green infrastructure approach."
**Liquete, C. K., S.: Dige, G.: Maes, J.: Grizzetti, B.: Olah, B.: Zulian, G.**	2015	Environmental Science & Policy	54	"Mapping green infrastructure based on ecosystem services and ecological networks: A Pan-European case study."
**Liu, L. F., O.: Zhang, S.**	2019	Water (Switzerland)	11(10)	"Blue-green infrastructure for sustainable urban stormwater management-lessons from six municipality-led pilot projects in Beijing and Copenhagen."
**Livingstone, S. W. C., M. W.: Isaac, M. E.**	2018	Ecosystem Services	30	"Ecological engagement determines ecosystem service valuation: A case study from Rouge National Urban Park in Toronto, Canada."
**Lopoukhine, N. C., N.: Dudley, N.: Figgis, P.: Karibuhoye, C.: Laffoley, D.: Miranda Londoño, J.: MacKinnon, K.: Sandwith, T.**	2012	Sapiens	5(2)	"Protected areas: Providing natural solutions to 21st Century challenges."
**Lovell, S. T. T., J. R.**	2013	Landscape Ecology	28(8)	"Supplying urban ecosystem services through multifunctional green infrastructure in the United States."
**Lynch, A. J.**	2016	Journal of Planning Education and Research	36(1)	"Is It Good to Be Green? Assessing the Ecological Results of County Green Infrastructure Planning."
**Mabelis, A. A. M., G.**	2009	International Journal of Biodiversity Science and Management	5(2)	"Public participation in green urban policy: Two strategies compared."
**MacIvor, J. S.**	2016	Israel Journal of Ecology & Evolution	62(1-2)	"Building height matters: nesting activity of bees and wasps on vegetated roofs."
**MacIvor, J. S. C., M. W.: Livingstone, S. W.: Lundholm, J. T.: Yasui, S. L. E.**	2016	Journal of Applied Ecology	53(5)	"Phylogenetic ecology and the greening of cities."
**MacIvor, J. S. M., L.: Puncher, C. L.: Carver Matthews, B. J.**	2013	Journal of Environmental Management	130	"Decoupling factors affecting plant diversity and cover on extensive green roofs."
**MacIvor, J. S. S., N.: Arnillas, C. A.: Bhatt, A.: Das, S.: Yasui, S. L. E.: Xie, G.: Cadotte, M. W.**	2018	Evolutionary Applications	11(10)	"Manipulating plant phylogenetic diversity for green roof ecosystem service delivery."
**Madre, F. V., A.: Machon, N.: Clergeau, P.**	2014	Landscape and Urban Planning	122	"Green roofs as habitats for wild plant species in urban landscapes: First insights from a large-scale sampling."
**Mancini, F. C., G. M.: Lusseau, D.**	2019	Journal of Applied Ecology	56(2)	"Quantifying wildlife watchers’ preferences to investigate the overlap between recreational and conservation value of natural areas."
**Manton, M. A., P.: Milberg, P.: Elbakidze, M.**	2016	Sustainability	8(4)	"Wet Grasslands as a Green Infrastructure for Ecological Sustainability: Wader Conservation in Southern Sweden as a Case Study."
**Martensson, L. M.**	2017	Ecological Engineering	103	"Methods of establishing species-rich meadow biotopes in urban areas."

Appendix S2. Included publications on green infrastructure and biodiversity

**Mathey, J. R., S.: Banse, J.: Lehmann, I.: Brauer, A.**	2015	Journal of Urban Planning and Development	141(3)	"Brownfields As an Element of Green Infrastructure for Implementing Ecosystem Services into Urban Areas."
**Matos, P. V., J.: Rocha, B.: Branquinho, C.: Pinho, P.**	2019	Science of the Total Environment	665	"Modeling the provision of air-quality regulation ecosystem service provided by urban green spaces using lichens as ecological indicators."
**Mattsson, B. J. V., H.**	2018	Conservation Biology	32(1)	"Prospects for stakeholder coordination by protected-area managers in Europe."
**Mayer-Pinto, M. J., E. L.: Bugnot, A. B.: Glasby, T. M.: Airoldi, L.: Mitchell, A.: Dafforn, K. A.**	2017	Journal of Environmental Management	189	"Building 'blue': An eco-engineering framework for foreshore developments."
**McWilliam, W. B., M.**	2017	Land Use Policy	68	"The role of dairy company policies in support of farm green infrastructure in the absence of government stewardship payments."
**Mell, I. C., S.**	2019	Impact Assessment and Project Appraisal		"Progressing Green Infrastructure planning: understanding its scalar, temporal, geo-spatial and disciplinary evolution."
**Merckx, T. V. D., H.**	2019	Global Ecology and Biogeography	28(10)	"Urbanization-driven homogenization is more pronounced and happens at wider spatial scales in nocturnal and mobile flying insects."
**Mikkonen, N. M., A.**	2013	Environmental Science & Policy	27	"Identification of top priority areas and management landscapes from a national Natura 2000 network."
**Mikolajczak, A. M., D.: Sanz, T.: Isenmann, M.: Thierion, V.: Luque, S.**	2015	Ecological Informatics	30	"Modelling spatial distributions of alpine vegetation: A graph theory approach to delineate ecologically-consistent species assemblages."
**Milanovich, J. R. P., W. E.: Barrett, K.: Hopton, M. E.**	2012	Landscape and Urban Planning	107(4)	"Do species distribution models predict species richness in urban and natural green spaces? A case study using amphibians."
**Molineux, C. J. G., A. C.: Connop, S. P.: Newport, D. J.**	2015	Ecological Engineering	82	"Using recycled aggregates in green roof substrates for plant diversity."
**Molineux, C. J. G., A. C.: Newport, D. J.**	2017	Science of the Total Environment	580	"Using soil microbial inoculations to enhance substrate performance on extensive green roofs."
**Mortberg, U. H., J.: Zetterberg, A.: Franklin, J. P.: Jonsson, D.: Deal, B.**	2013	Urban Ecosystems	16(4)	"Urban ecosystems and sustainable urban development-analysing and assessing interacting systems in the Stockholm region."
**Nilon, C. H. A., M. F. J.: Cilliers, S. S.: Dobbs, C.: Frazee, L. J.: Goddard, M. A.: O'Neill, K. M.: Roberts, D.: Stander, E. K.: Werner, P.: Winter, M.: Yocom, K. P.**	2017	Bioscience	67(4)	"Planning for the Future of Urban Biodiversity: A Global Review of City-Scale Initiatives."
**Norton, B. A. B., G. D.: Clark, R.: Corstanje, R.: Dunnett, N.: Evans, K. L.: Grafius, D. R.: Gravestock, E.: Grice, S. M.: Harris, J. A.: Hilton, S.: Hoyle, H.: Lim, E.: Mercer, T. G.: Pawlett, M.: Pescott, O. L.: Richards, J. P.: Southon, G. E.: Warren, P. H.**	2019	Ecological Applications	29(6)	"Urban meadows as an alternative to short mown grassland: effects of composition and height on biodiversity."
**Orsini, F. G., D.: Marchetti, L.: Piovene, C.: Draghetti, S.: Ramazzotti, S.: Bazzocchi, G.: Gianquinto, G.**	2014	Food Security	6(6)	"Exploring the production capacity of rooftop gardens (RTGs) in urban agriculture: the potential impact on food and nutrition security, biodiversity and other ecosystem services in the city of Bologna."
**Paal, T. K., L.: Lõhmus, K.: Liira, J.**	2017	Plant Ecology	218(4)	"Both spatiotemporal connectivity and habitat quality limit the immigration of forest plants into wooded corridors."

Appendix S2. Included publications on green infrastructure and biodiversity

**Palliwoda, J. K., I.: von der Lippe, M.**	2017	Landscape and Urban Planning	157	"Human-biodiversity interactions in urban parks: The species level matters."
**Papanikolaou, A. D. K., I.: Frenzel, M.: Schweiger, O.**	2017	Journal of Applied Ecology	54(2)	"Semi-natural habitats mitigate the effects of temperature rise on wild bees."
**Park, H. K., M.: Rhemtulla, J. M.: Konijnendijk, C. C.**	2019	Urban Forestry & Urban Greening	45	"Urban food systems that involve trees in Northern America and Europe: A scoping review."
**Pătru-Stupariu, I. A., P.: Elbakidze, M.: Huzui, A.: Andersson, K.**	2013	Biodiversity and Conservation	22(9)	"Using forest history and spatial patterns to identify potential high conservation value forests in Romania."
**Pelorosso, R. G., F.: Geri, F.: Leone, A.**	2017	Ecosystem Services	26	"PANDORA 3.0 plugin: A new biodiversity ecosystem service assessment tool for urban green infrastructure connectivity planning."
**Penone, C. M., N.: Julliard, R.: Le Viol, I.**	2012	Biological Conservation	148(1)	"Do railway edges provide functional connectivity for plant communities in an urban context?"
**Pétremand, G. C., Y.: Braaker, S.: Brenneisen, S.: Gerner, M.: Obrist, M. K.: Rochefort, S.: Szallies, A.: Moretti, M.**	2018	Urban Ecosystems	21(1)	"Ground beetle (Coleoptera: Carabidae) communities on green roofs in Switzerland: synthesis and perspectives."
**Petrisor, A. I. A., I. C.: Petrisor, L. E.: Ciobotaru, A. M.: Peptenatu, D. I. C. C. Ioja, L.: Dumitrache, L.: Nedelea, A.: Nita, M. R.**	2016	Ecosmart - Environment at Crossroads: Smart Approaches for a Sustainable Development.	32	Assessing the fragmentation of the green infrastructure in Romanian cities using fractal models and numerical taxonomy.
**Psaralexi, M. K. V., N. E. P.: Selva, N.: Mazaris, A. D.: Pantis, J. D.**	2017	Frontiers in Ecology and Evolution	5	"Importance of Roadless Areas for the European Conservation Network."
**Rae, M. M., A.: Hall, J.: O'Brien, K.: O'Brien, D.**	2019	Ecological Indicators	98	"Evaluating the validity of a simple citizen science index for assessing the ecological status of urban drainage ponds."
**Ramer, H. N., K. C.: Spivak, M.: Watkins, E.: Wolfin, J.: Pulscher, M.**	2019	Landscape and Urban Planning	189	"Exploring park visitor perceptions of 'flowering bee lawns' in neighborhood parks in Minneapolis, MN, US."
**Raymond, C. M. F., N.: Kabisch, N.: Berry, P.: Breil, M.: Nita, M. R.: Geneletti, D.: Calfapietra, C.**	2017	Environmental Science & Policy	77	"A framework for assessing and implementing the co-benefits of nature-based solutions in urban areas."
**Rega, C. B., A. M.: Bocci, G.: Sutter, L.: Albrecht, M.: Moonen, A. C.: Jeanneret, P.: van der Werf, W.: Pfister, S. C.: Holland, J. M.: Paracchini, M. L.**	2018	Ecological Indicators	90	"A pan-European model of landscape potential to support natural pest control services."
**Riley, C. B. H., D. A.: Gardiner, M. M.**	2018	Urban Forestry & Urban Greening	29	"Exotic trees contribute to urban forest diversity and ecosystem services in inner-city Cleveland, OH."
**Riley, C. B. P., K. I.: Ard, K.: Gardiner, M. M.**	2018	Sustainability (Switzerland)	10(7)	"Asset or liability? Ecological and sociological tradeoffs of urban spontaneous vegetation on vacant land in shrinking cities."
**Ristić, R. R., B.: Miljanović, V.: Trivan, G.: Ljujić, M.: Letić, L.: Savić, R.**	2013	Spatium	-30	"Blue-green corridors as a tool for mitigation of natural hazards and restoration of urbanized areas: A case study of belgrade city."
**Rodríguez-Loinaz, G. P., L.: Palacios-Agundez, I.: Ametzaga-Arregi, I.: Onaindia, M.**	2018	Forests	9(1)	"Identifying green infrastructure as a basis for an incentive mechanism at the municipality level in biscay (basque country)."
**Roeland, S. M., M.: Amorim, J. H.: Branquinho, C.: Fares, S.: Morelli, F.: Niinemets,: Paoletti, E.: Pinho, P.: Sgrigna, G.: Stojanovski, V.: Tiwary, A.: Sicard, P.: Calfapietra, C.**	2019	Journal of Forestry Research	30(6)	"Towards an integrative approach to evaluate the environmental ecosystem services provided by urban forest."
**Rolf, W. P., D.: Lenz, R.: Pauleit, S.**	2018	Ecological Indicators	94	"Farmland - an Elephant in the Room of Urban Green Infrastructure ? Lessons learned from connectivity analysis in three German cities."

Appendix S2. Included publications on green infrastructure and biodiversity

**Rudolph, M. V., F.: Schwenzfeier, S.: Kleinebecker, T.: Klaus, V. H.**	2017	Applied Vegetation Science	20(1)	"Patterns and potentials of plant species richness in high- and low-maintenance urban grasslands."
**Salomaa, A. P., R.: Kotiaho, J. S.: Kettunen, M.: Apostolopoulou, E.: Cent, J.**	2017	Environment and Planning C-Politics and Space	35(2)	"Can green infrastructure help to conserve biodiversity?"
**Sandström, U. G.**	2002	Planning Practice and Research	17(4)	"Green infrastructure planning in urban Sweden."
**Saumel, I. W., F.: Kowarik, I.**	2016	Environmental Science & Policy	62	"Toward livable and healthy urban streets: Roadside vegetation provides ecosystem services where people live and move."
**Saura, S. B., L.: Battistella, L.: Mandrici, A.: Dubois, G.**	2017	Ecological Indicators	76	"Protected areas in the world's ecoregions: How well connected are they?"
**Schindler, S. K., M.: Euller, K.: Bunting, S. W.: Schulz-Zunkel, C.: Hermann, A.: Hainz-Renetzeder, C.: Kanka, R.: Mauerhofer, V.: Gasso, V.: Krug, A.: Lauwaars, S. G.: Zulka, K. P.: Henle, K.: Hoffmann, M.: Biró, M.: Essl, F.: Jaquier, S.: Balázs, L.: Borics, G.: Hudin, S.: Damm, C.: Pusch, M.: Van Der Sluis, T.: Sebesvari, Z.: Wrbka, T.**	2013	Environmental Evidence	2(1)	"Floodplain management in temperate regions: Is multifunctionality enhancing biodiversity?"
**Schindler, S. O. N., F. H.: Biró, M.: Damm, C.: Gasso, V.: Kanka, R.: van der Sluis, T.: Krug, A.: Lauwaars, S. G.: Sebesvari, Z.: Pusch, M.: Baranovsky, B.: Ehlert, T.: Neukirchen, B.: Martin, J. R.: Euller, K.: Mauerhofer, V.: Wrbka, T.**	2016	Biodiversity and Conservation	25(7)	"Multifunctional floodplain management and biodiversity effects: a knowledge synthesis for six European countries."
**Schwarz, N. M., M.: Bugalho, M. N.: Davies, Z. G.: Haase, D.: Hack, J.: Hof, A.: Melero, Y.: Pett, T. J.: Knapp, S.**	2017	Ecosystem Services	27	"Understanding biodiversity-ecosystem service relationships in urban areas: A comprehensive literature review."
**Selim, S. S., N. K.: Onur, I.: Coslu, M.: Spie, C. M. U. M. Neale, A.**	2017	Remote Sensing for Agriculture, Ecosystems, and Hydrology Xix.	10421	Determination of the ecological connectivity between landscape patches obtained using the knowledge engineer (expert) classification technique.
**Shi, X. M. Q., M. Z.**	2018	Sustainability	10(12)	"Research on the Optimization of Regional Green Infrastructure Network."
**Shwartz, A. T., A.: Julliard, R.: Simon, L.: Prévot, A. C.**	2014	Global Environmental Change	28(1)	"Outstanding challenges for urban conservation research and action."
**Sikorska, D. S., P.: Archiciński, P.: Chormański, J.: Hopkins, R. J.**	2019	Sustainability (Switzerland)	11(20)	"You can't see the woods for the trees: Invasive Acer negundo L. in Urban riparian forests harms biodiversity and limits recreation activity."
**Sikorska, D. S., P.: Hopkins, R. J.**	2017	Sustainability	9(3)	"High Biodiversity of Green Infrastructure Does Not Contribute to Recreational Ecosystem Services."
**Simíc, I. S., A.: Djokíc, V.**	2017	Sustainability (Switzerland)	9(7)	"Building the green infrastructure of Belgrade: The importance of community greening."
**Sirakaya, A. C., A.: Harris, J.**	2018	Ecosystem Services	29	"Ecosystem services in cities: Towards the international legal protection of ecosystem services in urban environments."
**Sitzia, T. C., T.: Weir, R. G.**	2016	Urban Ecosystems	19(1)	"Novel woodland patches in a small historical Mediterranean city: Padova, Northern Italy."
**Snäll, T. L., J.: Arponen, A.: Elith, J.: Moilanen, A.**	2016	Environmental Management	57(2)	"Green Infrastructure Design Based on Spatial Conservation Prioritization and Modeling of Biodiversity Features and Ecosystem Services."
**Suchocka, M. B., M.: Juzwiak, A.: Duriasz, J.: Bohdan, A.: Stolarczyk, J.**	2019	Sustainability	11(6)	"Transit versus Nature. Depreciation of Environmental Values of the Road Alleys. Case Study: Gamerki-Jonkowo, Poland."

Appendix S2. Included publications on green infrastructure and biodiversity

**Svensson, J. A., J.: Sandström, P.: Mikusiński, G.: Jonsson, B. G.**	2019	Conservation Biology	33(1)	"Landscape trajectory of natural boreal forest loss as an impediment to green infrastructure."
**Talal, M. L. S., M. V.**	2019	Frontiers in Ecology and Evolution	7	"Plant Community Composition and Biodiversity Patterns in Urban Parks of Portland, Oregon."
**Threlfall, C. G. M., L.: Mackie, J. A.: Hahs, A. K.: Stork, N. E.: Williams, N. S. G.: Livesley, S. J.**	2017	Journal of Applied Ecology	54(6)	"Increasing biodiversity in urban green spaces through simple vegetation interventions."
**Tóth, A. K., G.: Feriancová, L.**	2016	Forestry Journal	62(1)	"Species composition and diversity of non-forest woody vegetation along roads in the agricultural landscape."
**Törnblom, J. A., P.: Degerman, E.: Tamario, C.**	2017	Ecoscience	24(3-4)	"Prioritizing Dam Removal and Stream Restoration Using Critical Habitat Patch Threshold for Brown Trout (Salmo trutta L.): A Catchment Case Study from Sweden."
**Vallecillo, S. P., C.: Barbosa, A.: Castillo, C. P.: Vandecasteele, I.: Rusch, G. M.: Maes, J.**	2018	Landscape and Urban Planning	174	"Spatial alternatives for Green Infrastructure planning across the EU: An ecosystem service perspective."
**van der Zanden, E. H. V., P. H.: Mucher, C. A.**	2013	Ecological Indicators	27	"Modelling the spatial distribution of linear landscape elements in Europe."
**Van Teeffelen, A. J. A. V., C. C.: Jochem, R.: Baveco, J. M.: Meeuwsen, H.: Hilbers, J. P.**	2015	Landscape Ecology	30(5)	"Is green infrastructure an effective climate adaptation strategy for conserving biodiversity? A case study with the great crested newt."
**Vannucchi, F. P., R.: Scatena, M.: Benelli, G.: Canale, A.: Bretzel, F.**	2018	Ecological Engineering	116	"Deinking sludge in the substrate reduces the fertility and enhances the plant species richness of extensive green roofs."
**Vanstockem, J. C., C.: Van Dyck, K.: Somers, B.: Hermy, M.**	2018	Applied Vegetation Science	21(3)	"Is there more than meets the eye? Seed bank analysis of a typical novel ecosystem, the extensive green roof."
**Vasiljevic, N. R., B.: Gavrilovic, S.: Sljukic, B.: Medarevic, M.: Ristic, R.**	2018	Iforest-Biogeosciences and Forestry	11	"The concept of green infrastructure and urban landscape planning: a challenge for urban forestry planning in Belgrade, Serbia."
**Verhagen, W. v. d. Z., E. H.: Strauch, M.: van Teeffelen, A. J. A.: Verburg, P. H.**	2018	Environmental Science & Policy	84	"Optimizing the allocation of agri-environment measures to navigate the trade-offs between ecosystem services, biodiversity and agricultural production."
**Verlic, A. D., N.: Kokalj, Z.: Marsetic, A.: Simoncic, P.: Ostir, K.**	2014	Sumarski List	138(9-10)	"TREE SPECIES CLASSIFICATION USING WORLDVIEW-2 SATELLITE IMAGES AND LASER SCANNING DATA IN A NATURAL URBAN FOREST."
**Villemey, A. J., A.: Vargac, M.: Bertheau, Y.: Coulon, A.: Touroult, J.: Vanpeene, S.: Castagneyrol, B.: Jactel, H.: Witte, I.: Deniaud, N.: De Lachapelle, F. F.: Jaslier, E.: Roy, V.: Guinard, E.: Le Mitouard, E.: Rauel, V.: Sordello, R.**	2018	Environmental Evidence	7(1)	"Can linear transportation infrastructure verges constitute a habitat and/or a corridor for insects in temperate landscapes? A systematic review."
**Wamsler, C. N., L.: Beery, T. H.: Bramryd, T.: Ekelund, N.: Jönsson, K. I.: Osmani, A.: Palo, T.: Stålhammar, S.**	2016	Ecology and Society	21(1)	"Operationalizing ecosystem-based adaptation: Harnessing ecosystem services to buffer communities against climate change."
**Wang, H. F. Q., S.: Knapp, S.: Friedman, C. R.: Hubacek, K.**	2015	Applied Geography	64	"A basic assessment of residential plant diversity and its ecosystem services and disservices in Beijing, China."
**Ware, J. C., R.**	2019	PLoS ONE	14(10)	"Public perception of coastal habitat loss and habitat creation using artificial floating islands in the UK."
**Weber, T. C. B., P. J.: Sloan, A.**	2008	Environmental Management	41(4)	"Field validation of a conservation network on the eastern shore of Maryland, USA, using breeding birds as bio-indicators."

Appendix S2. Included publications on green infrastructure and biodiversity

**Weber, T. S., A.: Wolf, J.**	2006	Landscape and Urban Planning	77(1-2)	"Maryland's Green Infrastructure Assessment: Development of a comprehensive approach to land conservation."
**Williams, N. S. G. L., J.: Scott Macivor, J.**	2014	Journal of Applied Ecology	51(6)	"Do green roofs help urban biodiversity conservation?"
**Wirth, P. C., J.: Syrbe, R. U.: Wende, W.: Hu, T.**	2018	International Journal of Coal Science and Technology	5(1)	"Green infrastructure: a planning concept for the urban transformation of former coal-mining cities."
**Wong, G. K. L. J., C. Y.**	2016	Science of the Total Environment	573	"Do vegetated rooftops attract more mosquitoes? Monitoring disease vector abundance on urban green roofs."
**Xie, C. P.**	2018	Fresenius Environmental Bulletin	27(12A)	"TREE DIVERSITY IN URBAN PARKS OF DUBLIN, IRELAND."
**Xu, H. Y. P., T.: Primdahl, J.**	2019	Land	8(3)	"A Systematic Comparison of Cultural and Ecological Landscape Corridors in Europe."
**Yamanaka, S. I., N.: Senzaki, M.: Morimoto, J.: Kitazawa, M.: Fuke, N.: Nakamura, F.**	2020	Ecological Engineering	142	"Role of flood-control basins as summer habitat for wetland species - A multiple-taxon approach."
**Zhang, D. W., W.: Zheng, H.: Ren, Z.: Zhai, C.: Tang, Z.: Shen, G.: He, X.**	2017	Ecological Indicators	80	"Effects of urbanization intensity on forest structural-taxonomic attributes, landscape patterns and their associations in Changchun, Northeast China: Implications for urban green infrastructure planning."
**Zhang, D. Z., H.: He, X.: Ren, Z.: Zhai, C.: Yu, X.: Mao, Z.: Wang, P.**	2016	Urban Ecosystems	19(1)	"Effects of forest type and urbanization on species composition and diversity of urban forest in Changchun, Northeast China."
**Zhang, Z. M., S.: Newell, J. P.: Lindquist, M.**	2019	Urban Forestry and Urban Greening	38	"Enhancing landscape connectivity through multifunctional green infrastructure corridor modeling and design."
**Ziter, C.**	2016	Oikos	125(6)	"The biodiversity-ecosystem service relationship in urban areas: a quantitative review."
**Zmelik, K. S., S.: Wrbka, T.**	2011	Innovation-the European Journal of Social Science Research	24(3)	"The European Green Belt: international collaboration in biodiversity research and nature conservation along the former Iron Curtain."

Appendix S3. Metadata collected for scientific publications on ecological processes

MAIN CATEGORIES	DATA NOTED	EXPLANAITION
**ID**		ID for this study
**AUTHOR**		From endnote
**YEAR**		From endnote
**TITLE**		From endnote
**JOURNAL**		From endnote
**VOLUME:ISSUE**		From endnote
**PAGES**		From endnote
**ABSTRACT**		From endnote
**REASON FOR INCLUSION**	Ecological process 1/0	Should relate to ecological and demographic processes at species/population level, excluding biodiversity patterns at community level (diversity/richness). Processes are specified within search string to be; survival, reproduction, dispersal, immigration, emigration, extinction, colonisation, recolonisation, population demography, mortality, productivity. Write 1 if this applies to the study, 0 if not.
**REASON FOR INCLUSION**	Habitat attribute1/0	Paper should relate results to habitat attributes defined in search string; habitat area, quantity, connectivity, isolation, structure, aggregation, fragmentation, configuration, amount. Quality is only relevant in combination with other attributes, not patches within a habitat (scale). Write 1 if this applies to the study, 0 if not.
**REASON FOR INCLUSION**	GI context 1/0	Paper should be relevant for the GI context (landscape perspective) defined in this review; not marine, at least partly terrestrial, mainland oriented and studies only interesting in relation to a specified spatial context and ecological process. Write 1 if this applies to the study, 0 if not.
**SCALES**	too small/relevant/too big/not relevant	Is the scale relevant for the GI-concept? Too small scales acting within habitat patches is not relevant if not in relation to other habitats. Too big continental scale is not relevant either. Time scale is also considered here, historical patterns not of interest. Not relevant refers to studies where the scale is of non- importance. Put scale even if other criteria are not fulfilled.

Appendix S3. Metadata collected for scientific publications on ecological processes

**INCLUSION**	y/n	Put y if all three reasons for inclusion are met (not scale), put n if one or more is missing. Put maybe if not possible to determine from title and abstract but try to avoid since this means full-text is necessary. If maybe, leave the uncertain inclusion critera blank and write comment in column R.
**EXCLUSION**	Put missing inclusion term	Give the first missing inclusion term
**TYPE OF ARTICLE**	Empirical	Is this an empirical study or is it theoretical or conceptual. This determines if it is relevant for a review or discussion.
**TYPE OF ARTICLE**	Review	Is this a compilation of studies, a review on a topic of relevance for this study. Determines if this is relevant for a review or for a discussion. Put 1/0.
**RESULTS**	Response variable	Put the first response variable reported in the study, enter additional response variables on separate rows. Put separate species on separate rows.
**RESULTS**	Process reported	Put the process reported, survival, dispersal, mortality, productivity etc. If not explicitly referred to, leave blank.
**RESULTS**	Habitat attribute	In relation to what habitat attribute is the response variable analysed. In relation to increasing habitat attribute variable. Population size effects are only entered here if they are analysed besides other spatially explicit variables. If population size is the habitat attribute referred to the result is not included.
**RESULTS**	Direction of effects	In what direction of effects for the habitat attribute on the response variable. Put p for positive, n for negative, 0 for non-significant result and ? For non-conclusive/inconclusive.
**DOMINATING LAND COVER (LUCAS)**	Put as specified according to LUCAS; artificial land;cropland; woodland;shrubland; grassland;bare land and lichens/moss;inland water;wetland	Put term of the dominating land cover described
**LAND COVER AUTHOR**	Put term	Put the term used by the author, could be other than LUCAS
**PREFERRED HABITAT (LUCAS)**	Put as specified according to LUCAS; artificial land;cropland; woodland;shrubland;	Put term of the dominating land cover described

Appendix S3. Metadata collected for scientific publications on ecological processes

	grassland;bare land and lichens/moss;inland water;wetland	
**PREFERRED HABITAT AUTHOR**	Put term	Put the term used by the author, could be other than LUCAS
**TAXONOMIC CLASSIFICATION**	mammals;birds;reptiles; amphibians;insects;spiders; vascular plants;non vascular plants;fungi	Put classification as specified in table

Appendix S4. Included publications in review of effects from habitat features, sorted by relevant ecological process.

PROCESS	AUTHOR	JOURNAL	YEAR
**COLONIZATION**	Elzinga, J. A., Van Nouhuys, S., Van Leeuwen, D. J. and Biere, A.	Basic and Applied Ecology	2007
	Crooks, K. R., Suarez, A. V., Bolger, D. T. and Soule, M. E.	Conservation Biology	2001
	Sebastián-González, E., Alexander, K. L., Sánchez-Zapata, J. A. and Botella, F.	Ardeola	2015
	Scholtz, R., Polo, J. A., Fuhlendorf, S. D. and Duckworth, G. D.	Biological Conservation	2017
	Zartman, C. E. and Jonathan Shaw, A.	American Naturalist	2006
	Woodcock, Ben A., Vogiatzakis, Ioannis N., Westbury, Duncan B., Lawson, Clare S., Edwards, Andrew R., Brook, Alex J., Harris, Stephanie J., Lock, Kerry A., Maczey, Norbert, Masters, Greg, Brown, Valerie K. and Mortimer, Simon R.	Journal of Applied Ecology	2010
	Magle, S. B., Lehrer, E. W. and Fidino, M.	Animal Conservation	2016
	Weterings, Robbie, Umponstira, Chanin and Buckley, Hannah L.	Basic and Applied Ecology	2014
	Sartain, Autumn R. and Alberts, Allison C.	Western Birds	2008
	Holland, Greg J. and Bennett, Andrew F.	Austral Ecology	2011
	Yackulic, C. B., Reid, J., Davis, R., Hines, J. E., Nichols, J. D. and Forsman, E.	Ecology	2012
	Yao, J., Holt, R. D., Rich, P. M. and Marshall, W. S.	Ecography	1999
	Stouffer, Philip C., Johnson, Erik I., Bierregaard, Richard O., Jr. and Lovejoy, Thomas E.	Plos One	2011
	Mason, C. F.	Biodiversity and Conservation	2001
	Bruna, E. M., Vasconcelos, H. L. and Heredia, S.	Biological Conservation	2005
	Fourcade, Yoan, Ranius, Thomas and Ockinger, Erik	Journal of Animal Ecology	2017
	Hames, R. S., Rosenberg, K. V., Lowe, J. D. and Dhondt, A. A.	Journal of Animal Ecology	2001
	Marsh, D. M.	Ecology	2001
	Schooley, R. L. and Branch, L. C.	Ecological Applications	2009
	Thomas, C. D. and Jones, T. M.	Journal of Animal Ecology	1993
	Chavel, Emilie E., Imbeau, Louis, Mazerolle, Marc J. and Drapeau, Pierre	Forest Ecology and Management	2017
	Klapwijk, Maartje J. and Lewis, Owen T.	Basic and Applied Ecology	2012
	Beasley, James C., Olson, Zachary H., Beatty, William S., Dharmarajan, Guha and Rhodes, Olin E., Jr.	Plos One	2013

Appendix S4. Included publications in review of effects from habitat features, sorted by relevant ecological process.

	Berggren, A., Carlson, A. and Kindvall, O.	Journal of Animal Ecology	2001
	Anadon, J. D., Perez-Garcia, J. M., Perez, I., Royo, J. and Sanchez-Zapata, J. A.	Landscape Ecology	2018
	Brown, Jessi L., Collopy, Michael W. and Smallwood, John A.	Bird Conservation International	2014
	Cosentino, Bradley J., Schooley, Robert L. and Phillips, Christopher A.	Landscape Ecology	2010
	Collinge, S. K., Holyoak, M., Barr, C. B. and Marty, J. T.	Biological Conservation	2001
	Binzenhoefer, Birgit, Biedermann, Robert, Settele, Josef and Schroeder, Boris	Ecological Research	2008
	Crampton, Lisa H., Longland, William S., Murphy, Dennis D. and Sedinger, James S.	Oikos	2011
	Bowne, D. R., Peles, J. D. and Barrett, G. W.	Landscape Ecology	1999
**DISPERSAL**	Zheng, Chaozhi, Ovaskainen, Otso and Hanski, Ilkka	Philosophical Transactions of the Royal Society B-Biological Sciences	2009
	Stephens, Helen C., Schmuki, Christina, Burridge, Christopher P. and O'reilly-Wapstra, Julianne M.	Austral Ecology	2013
	Guivier, E., Galan, M., Chaval, Y., Xuereb, A., Ribas Salvador, A., Poulle, M. L., Voutilainen, L., Henttonen, H., Charbonnel, N. and Cosson, J. F.	Molecular Ecology	2011
	Severns, Paul M., Mcintire, Eliot J. B. and Schultz, Cheryl B.	Landscape Ecology	2013
	Cascante, A., Quesada, M., Lobo, J. J. and Fuchs, E. A.	Conservation Biology	2002
	Lancaster, Melanie L., Taylor, Andrea C., Cooper, Steven J. B. and Carthew, Susan M.	Molecular Ecology	2011
	Mimura, Makiko, Barbour, Robert C., Potts, Brad M., Vaillancourt, Rene E. and Watanabe, Kazuo N.	Molecular Ecology	2009
	De La Pena-Domene, Marinrs, Minor, Emily S. and Howe, Henry F.	Ecology	2016
	Damschen, Ellen I., Baker, Dirk V., Bohrer, Gil, Nathan, Ran, Orrock, John L., Turner, Jay R., Brudvig, Lars A., Haddad, Nick M., Levey, Douglas J. and Tewksbury, Joshua J.	Proceedings of the National Academy of Sciences of the United States of America	2014
	Farwig, Nina, Schabo, Dana G. and Albrecht, Jorg	Journal of Ecology	2017
	Coulon, Aurelie, Fitzpatrick, John W., Bowman, Reed and Lovette, Irby J.	Biology Letters	2012
	Bruna, E. M.	Ecology	2003
	Ryan, M. R., Burger, L. W., Jones, D. P. and Wywialowski, A. P.	American Midland Naturalist	1998
	Ismail, S. A., Ghazoul, J., Ravikanth, G., Shaanker, R. Uma, Kushalappa, C. G. and Kettle, C. J.	Molecular Ecology	2012

Appendix S4. Included publications in review of effects from habitat features, sorted by relevant ecological process.

	Guiden, Peter W.	Journal of the Torrey Botanical Society	2017
	Haddad, N. M. and Tewksbury, J. J.	Ecological Applications	2005
	Xue, Huiliang, Zhong, Min, Xu, Jinhui and Xu, Laixiang	Plos One	2014
	Ismail, S. A., Ghazoul, J., Ravikanth, G., Kushalappa, C. G., Shaanker, R. Uma and Kettle, C. J.	Conservation Genetics	2014
	Clemencet, J., Viginier, B. and Doums, C.	Molecular Ecology	2005
	Balbi, Manon, Ernoult, Aude, Poli, Pedro, Madec, Luc, Guiller, Annie, Martin, Marie-Claire, Nabucet, Jean, Beaujouan, Veronique and Petit, Eric J.	Molecular Ecology	2018
	Maag, Nino, Karpati, Theresa and Bollmann, Kurt	Biological Conservation	2013
	Stevens, Kate P., Harrisson, Katherine A., Clarke, Rohan H., Cooke, Raylene and Hogan, Fiona E.	Emu	2016
	Cheptou, P. O., Carrue, O., Rouifed, S. and Cantarel, A.	Proceedings of the National Academy of Sciences of the United States of America	2008
	Anderson, Sara J., Kierepka, Elizabeth M., Swihart, Robert K., Latch, Emily K. and Rhodes, Olin E., Jr.	Plos One	2015
	Van Geert, Anja, Van Rossum, Fabienne and Triest, Ludwig	Journal of Ecology	2010
	Martins, Karina, Kimura, Renato Kenji, Francisconi, Ana Flavia, Gezan, Salvador, Kainer, Karen and Christianini, Alexander V.	Conservation Genetics	2016
	Naoe, Shoji, Sakai, Shoko, Sawa, Ayako and Masaki, Takashi	Ecological Research	2011
	Nagamitsu, Teruyoshi, Kikuchi, Satoshi, Hotta, Mayuko, Kenta, Tanaka and Hiura, Tsutom	American Midland Naturalist	2014
	Lawes, Timothy J., Anthony, Robert G., Robinson, W. Douglas, Forbes, James T. and Lorton, Glenn A.	Western North American Naturalist	2012
	Komuro, T. and Koike, F.	Ecological Applications	2005
	Santos, T., Telleria, J. L. and Virgos, E.	Ecography	1999
	Van Rossum, Fabienne and Triest, Ludwig	Landscape and Urban Planning	2010
	Wolf, A.	Biological Conservation	2001
	Vandewoestijne, Sofie, Schtickzelle, Nicolas and Baguette, Michel	Bmc Biology	2008
	Lehouck, V., Spanhove, T., Colson, L., Adringa-Davis, A., Cordeiro, N. J. and Lens, L.	Oikos	2009

Appendix S4. Included publications in review of effects from habitat features, sorted by relevant ecological process.

Chiappero, Marina B., Sommaro, Lucia V., Priotto, Jose W., Paula Wiernes, Maria, Steinmann, Andrea R. and Gardenal, Cristina N.	Journal of Mammalogy	2016
Pavlova, Alexandra, Amos, J. Nevil, Goretskaia, Maria I., Beme, Irina R., Buchanan, Katherine L., Takeuchi, Naoko, Radford, James Q. and Sunnucks, Paul	Ecology	2012
Uriarte, Maria, Anciaes, Marina, Da Silva, Mariana T. B., Rubim, Paulo, Johnson, Erik and Bruna, Emilio M.	Ecology	2011
Serio-Silva, J. C. and Rico-Gray, V.	Oryx	2002
Mceuen, Amy B. and Curran, Lisa M.	Plant Ecology	2006
Fischer, Marietta L., Sullivan, Martin J. P., Greiser, Grit, Guerrero-Casado, Jose, Heddergott, Mike, Hohmann, Ulf, Keuling, Oliver, Lang, Johannes, Martin, Ina, Michler, Frank-Uwe, Winter, Armin and Klein, Roland	Biological Invasions	2016
Diaz, M., Santos, T. and Telleria, J. L.	Acta Oecologica-International Journal of Ecology	1999
Currie, D. and Matthysen, E.	Belgian Journal of Zoology	1998
Gabrielsen, Charlotte G., Kovach, Adrienne I., Babbitt, Kimberly J. and Mcdowell, William H.	Conservation Genetics	2013
Umapathy, G. and Kumar, A.	Primates	2000
Herrera, J. M., De Sá Teixeira, I., Rodríguez-Pérez, J. and Mira, A.	Landscape Ecology	2016
Matthysen, E.	Oecologia	1999
Taylor, Andrea C., Tyndale-Biscoe, Hugh and Lindenmayer, David B.	Molecular Ecology	2007
Parejo-Farnes, Clara, Robledo-Arnuncio, Juan J., Albaladejo, Rafael G., Rubio-Perez, Encarnacion and Aparicio, Abelardo	Tree Genetics & Genomes	2017
Epps, Clinton W., Palsboll, Per J., Wehausen, John D., Roderick, George K. and Mccullough, Dale R.	Molecular Ecology	2006
Rosa, Jaqueline Figueredo, Ramalho, Mauro and Arias, Maria Cristina	Biotropica	2016
Garcia, Daniel, Martinez, Daniel, Herrera, Jose M. and Morales, Juan M.	Ecography	2013
Dileo, M. F., Rico, Y., Boehmer, H. J. and Wagner, H. H.	Biological Conservation	2017
Harrisson, Katherine A., Pavlova, Alexandra, Amos, J. Nevil, Radford, James Q. and Sunnucks, Paul	Journal of Animal Ecology	2014
Lada, Hania, Thomson, James R., Mac Nally, Ralph and Taylor, Andrea C.	Journal of Applied Ecology	2008
Jacquemyn, Hans, Vandepitte, Katrien, Brys, Rein, Honnay, Olivier and Roldan-Ruiz, Isabel	Biological Conservation	2007
Cortes, Marina C., Uriarte, Maria, Lemes, Maristerra R., Gribel, Rogerio, Kress, W. John, Smouse, Peter E. and Bruna, Emilio M.	Molecular Ecology	2013
Masaki, T.	Plant Ecology	2004

Appendix S4. Included publications in review of effects from habitat features, sorted by relevant ecological process.

Ney, Gideon and Schul, Johannes	Conservation Genetics	2017
Richmond, Jonathan Q., Reid, Duncan T., Ashton, Kyle G. and Zamudio, Kelly R.	Conservation Genetics	2009
Watts, P. C., Saccheri, I. J., Kemp, S. J. and Thompson, D. J.	Freshwater Biology	2006
Lecomte, J., Boudjemadi, K., Sarrazin, F., Cally, K. and Clobert, J.	Journal of Animal Ecology	2004
Vignieri, S. N.	Molecular Ecology	2005
Thiele, Jan, Schuckert, Ulrike and Otte, Annette	Landscape Ecology	2008
Evans, D. M., Levey, D. J. and Tewksbury, J. J.	Ecological Restoration	2013
Cornelius, Cintia, Awade, Marcelo, Candia-Gallardo, Carlos, Sieving, Kathryn E. and Metzger, Jean Paul	Perspectives in Ecology and Conservation	2017
Van Rossum, Fabienne, Leprince, Nathalie, Mayer, Carolin, Raabova, Jana, Hans, Guillaume and Jacquemart, Anne-Laure	Plant Ecology and Evolution	2015
Stanton, Sharon, Honnay, Olivier, Jacquemyn, Hans and Roldan-Ruiz, Isabel	Plant Systematics and Evolution	2009
Ratkiewicz, Miroslaw, Matosiuk, Maciej, Saveljev, Alexander P., Sidorovich, Vadim, Ozolins, Janis, Maennil, Peep, Balciauskas, Linas, Kojola, Ilpo, Okarma, Henryk, Kowalczyk, Rafal and Schmidt, Krzysztof	Plos One	2014
Millar, Melissa A., Coates, David J. and Byrne, Margaret	Annals of Botany	2014
Scherer, Rick D., Muths, Erin, Noon, Barry R. and Oyler-Mccance, Sara J.	Conservation Genetics	2012
Farwig, N., Bohning-Gaese, K. and Bleher, B.	Oecologia	2006
Fuchs, E. J., Lobo, J. A. and Quesada, M.	Conservation Biology	2003
Sullivan, Lauren L., Johnson, Brenda L., Brudvig, Lars A. and Haddad, Nick M.	Ecology	2011
Bergman, K. O. and Landin, J.	Biological Conservation	2001
Purrenhage, J. L., Niewiarowski, P. H. and Moore, F. B. G.	Molecular Ecology	2009
Row, Jeffrey R., Blouin-Demers, Gabriel and Lougheed, Stephen C.	Molecular Ecology	2010
Belletti, P., Monteleone, I. and Ferrazzini, D.	European Journal of Forest Research	2008
Yineger, Haile, Schmidt, Daniel J., Teketay, Demel, Zalucki, Jacinta and Hughes, Jane M.	Biological Journal of the Linnean Society	2015
Mora, Matias S., Mapelli, Fernando J., Gaggiotti, Oscar E., Kittlein, Marcelo J. and Lessa, Enrique P.	BMC Genetics	2010
Cramer, J. M., Mesquita, R. C. G. and Bruce Williamson, G.	Biological Conservation	2007
Macqueen, P. E., Nicholls, J. A., Hazlitt, S. L. and Goldizen, A. W.	Austral Ecology	2008
Barnett, Jacob R., Ruiz-Gutierrez, Viviana, Coulon, Aurelie and Lovette, Irby J.	Conservation Genetics	2008

Appendix S4. Included publications in review of effects from habitat features, sorted by relevant ecological process.

Herrera-Arroyo, M. L., Sork, V. L., González-Rodríguez, A., Rocha-Ramírez, V., Vega, E. and Oyama, K.	American Journal of Botany	2013
Winkler, Manuela, Koch, Marcus and Hietz, Peter	Conservation Genetics	2011
Berkman, Leah K., Nielsen, Clayton K., Roy, Charlotte L. and Heist, Edward J.	Conservation Genetics	2013
Born, C., Hardy, O. J., Chevallier, M. H., Ossari, S., Attéké, C., Wickings, E. J. and Hossaert-Mckey, M.	Molecular Ecology	2008
Head, Jennifer R., Chang, Howard, Li, Qunna, Hoover, Christopher M., Wilke, Thomas, Clewing, Catharina, Carlton, Elizabeth J., Liang, Song, Lu, Ding, Zhong, Bo and Remais, Justin V.	Plos Neglected Tropical Diseases	2016
Crawford, John A., Peterman, William E., Kuhns, Andrew R. and Eggert, Lori S.	Landscape Ecology	2016
Castilla, Antonio R., Pope, Nathaniel, Jaffe, Rodolfo and Jha, Shalene	Plos One	2016
Berge, G., Nordal, I. and Hestmark, G.	Oikos	1998
Banks, S. C., Ward, S. J., Lindenmayer, D. B., Finlayson, G. R., Lawson, S. J. and Taylor, A. C.	Molecular Ecology	2005
Clark, Joseph D., Laufenberg, Jared S., Davidson, Maria and Murrow, Jennifer L.	Journal of Wildlife Management	2015
Aavik, Tsipe, Holderegger, Rolf, Edwards, Peter J. and Billeter, Regula	Journal of Applied Ecology	2013
Braga, A. C. and Collevatti, R. G.	Heredity	2011
Aben, Job, Bocedi, Greta, Palmer, Stephen C. F., Pellikka, Petri, Strubbe, Diederik, Hallmann, Caspar, Travis, Justin M. J., Lens, Luc and Matthysen, Erik	Journal of Applied Ecology	2016
Bonelli, Simona, Vrabec, Vladimir, Witek, Magdalena, Barbero, Francesca, Patricelli, Dario and Nowicki, Piotr	Population Ecology	2013
Banks, S. C., Finlayson, G. R., Lawson, S. J., Lindenmayer, D. B., Paetkau, D., Ward, S. J. and Taylor, A. C.	Biological Conservation	2005
Angold, P. G., Sadler, J. P., Hill, M. O., Pullin, A., Rushton, S., Austin, K., Small, E., Wood, B., Wadsworth, R., Sanderson, R. and Thompson, K.	Science of the Total Environment	2006
Bowman, J. and Fahrig, L.	Canadian Journal of Zoology-Revue Canadienne De Zoologie	2002
Berggren, A., Carlson, A. and Kindvall, O.	Journal of Animal Ecology	2001
Blair, Mary E. and Melnick, Don J.	Plos One	2012
Cordeiro, N. J. and Howe, H. F.	Proceedings of the National Academy of Sciences of the United States of America	2003
Bani, L., Orioli, V., Pisa, G., Fagiani, S., Dondina, O., Fabbri, E., Randi, E., Sozio, G. and Mortelliti, A.	Conservation Genetics	2017

Appendix S4. Included publications in review of effects from habitat features, sorted by relevant ecological process.

	Boccaccio, Luigi and Petacchi, Ruggero	Biocontrol	2009
	Collevatti, Rosane G., Telles, Mariana P. C., Lima, Jacqueline S., Gouveia, Felipe O. and Soares, Thannya N.	Plant Systematics and Evolution	2014
	Chen, Qiong, Tomlinson, Kyle W., Cao, Lin and Wang, Bo	Integrative Zoology	2017
	Alberto Puig-Lagunes, Angel, Canales-Espinosa, Domingo, Rangel-Negrin, Ariadna and Dias, Pedro Americo D.	International Journal of Primatology	2016
	Arroyo-Rodríguez, V., Aguilar-Barajas, E., González-Zamora, A., Rocha-Ramírez, V., González-Rodríguez, A. and Oyama, K.	Journal of Tropical Ecology	2017
	Cramer, J. M., Mesquita, R. C. G., Bentos, T. V., Moser, B. and Williamson, G. B.	Biotropica	2007
	Bosschieter, L., Goedhart, P. W., Foppen, R. P. B. and Vos, C. C.	Ardea	2010
	Cordeiro, Norbert J., Ndangalasi, Henry J., Mcentee, Jay P. and Howe, Henry F.	Ecology	2009
	Blair, C., Jiménez Arcos, V. H., De La Cruz, F. R. M. and Murphy, R. W.	Conservation Genetics	2015
	Andreazzi, C. S., Pimenta, C. S., Pires, A. S., Fernandez, F. A. S., Oliveira-Santos, L. G. and Menezes, J. F. S.	Biotropica	2012
	Bonte, Dries, Vanden Borre, Jeroen, Lens, Luc and Maelfait, Jean-Pierre	Animal Behaviour	2006
	Armstrong, D. P., Mcarthur, N., Govella, S., Morgan, K., Johnston, R., Gorman, N., Pike, R. and Richard, Y.	Biological Conservation	2013
	Chaves, Oscar M., Stoner, Kathryn E., Arroyo-Rodriguez, Victor and Estrada, Alejandro	International Journal of Primatology	2011
	Bossart, J. L. and Antwi, Josephine B.	Conservation Genetics	2013
	Coulon, A., Cosson, J. F., Angibault, J. M., Cargnelutti, B., Galan, M., Morellet, N., Petit, E., Aulagnier, S. and Hewison, A. J. M.	Molecular Ecology	2004
	Coulon, Aurelie, Fitzpatrick, John W., Bowman, Reed and Lovette, Irby J.	Conservation Biology	2010
	Andrianarimisa, A., Bachmann, L., Ganzhorn, J. U., Goodman, S. M. and Tomiuk, J.	Journal of Ornithology	2000
	Browne, L., Ottewell, K. and Karubian, J.	Heredity	2015
	Bowne, D. R., Peles, J. D. and Barrett, G. W.	Landscape Ecology	1999
**EMIGRATION**	G. Rabasa, S., Gutiérrez, D. and Escudero, A.	Oikos	2007
	Matter, S. F., Roland, J., Keyghobadi, N. and Sabourin, K.	American Midland Naturalist	2003
	Delattre, Thomas, Vernon, Philippe and Burel, Francoise	Agriculture Ecosystems & Environment	2013
	Cornelius, Cintia, Awade, Marcelo, Candia-Gallardo, Carlos, Sieving, Kathryn E. and Metzger, Jean Paul	Perspectives in Ecology and Conservation	2017

Appendix S4. Included publications in review of effects from habitat features, sorted by relevant ecological process.

	Anderson, Christine S. and Meikle, Douglas B.	Conservation Genetics	2010
	Bergman, K. O. and Landin, J.	Biological Conservation	2001
	Alderman, J., Mccollin, D., Hinsley, S. A., Bellamy, P. E., Picton, P. and Crockett, R.	Landscape Ecology	2005
	Bonelli, Simona, Vrabec, Vladimir, Witek, Magdalena, Barbero, Francesca, Patricelli, Dario and Nowicki, Piotr	Population Ecology	2013
	Akeboshi, A., Takagi, S., Murakami, M., Hasegawa, M. and Miyashita, T.	Journal of Insect Conservation	2015
	Baguette, M., Mennechez, G., Petit, S. and Schtickzelle, N.	Comptes Rendus Biologies	2003
	Banks, Sam C. and Lindenmayer, David B.	Journal of Animal Ecology	2014
	Didham, R. K., Lawton, J. H., Hammond, P. M. and Eggleton, P.	Philosophical Transactions of the Royal Society B-Biological Sciences	1998
	Day, J. R. and Possingham, H. P.	Theoretical Population Biology	1995
	Leon-Cortes, J. L., Lennon, J. J. and Thomas, C. D.	Oikos	2003
	Crooks, K. R., Suarez, A. V., Bolger, D. T. and Soule, M. E.	Conservation Biology	2001
	Sebastián-González, E., Alexander, K. L., Sánchez-Zapata, J. A. and Botella, F.	Ardeola	2015
	Scholtz, R., Polo, J. A., Fuhlendorf, S. D. and Duckworth, G. D.	Biological Conservation	2017
	Zartman, C. E. and Jonathan Shaw, A.	American Naturalist	2006
	Rannap, Riinu, Lohmus, Asko and Jakobson, Kaidi	Wetlands	2007
	Magle, S. B., Lehrer, E. W. and Fidino, M.	Animal Conservation	2016
	Sartain, Autumn R. and Alberts, Allison C.	Western Birds	2008
	Voegeli, Matthias, Serrano, David, Pacios, Fernando and Tella, Jose L.	Biological Conservation	2010
	Yackulic, C. B., Reid, J., Davis, R., Hines, J. E., Nichols, J. D. and Forsman, E.	Ecology	2012
	Williams, Nicholas S. G., Morgan, John W., Mccarthy, Michael A. and Mcdonnell, Mark J.	Ecology	2006
	Ferraz, G., Russell, G. J., Stouffer, P. C., Bierregaard, R. O., Pimm, S. L. and Lovejoy, T. E.	Proceedings of the National Academy of Sciences of the United States of America	2003
	Krauss, J., Steffan-Dewenter, I. and Tscharntke, T.	Oecologia	2003
	Stouffer, Philip C., Johnson, Erik I., Bierregaard, Richard O., Jr. and Lovejoy, Thomas E.	Plos One	2011

Appendix S4. Included publications in review of effects from habitat features, sorted by relevant ecological process.

	Fourcade, Yoan, Ranius, Thomas and Ockinger, Erik	Journal of Animal Ecology	2017
	Rushton, S. P., Barreto, G. W., Cormack, R. M., Macdonald, D. W. and Fuller, R.	Journal of Applied Ecology	2000
	Bar-David, S., Segev, O., Peleg, N., Hill, N., Templeton, A. R., Schultz, C. B. and Blaustein, L.	Israel Journal of Ecology and Evolution	2007
	Hames, R. S., Rosenberg, K. V., Lowe, J. D. and Dhondt, A. A.	Journal of Animal Ecology	2001
	Elderd, B. D. and Nott, M. Philip	Journal of Applied Ecology	2008
	Schooley, R. L. and Branch, L. C.	Ecological Applications	2009
	Thomas, C. D. and Jones, T. M.	Journal of Animal Ecology	1993
	Chavel, Emilie E., Imbeau, Louis, Mazerolle, Marc J. and Drapeau, Pierre	Forest Ecology and Management	2017
	Cosentino, Bradley J., Schooley, Robert L. and Phillips, Christopher A.	Landscape Ecology	2010
	Collinge, S. K., Holyoak, M., Barr, C. B. and Marty, J. T.	Biological Conservation	2001
	Avila-Flores, R., Ceballos, G., Villa-Meza, A. D., List, R., Marcé, E., Pacheco, J., Arturo Sánchez-Azofeifa, G. and Boutin, S.	Biological Conservation	2012
	Crampton, Lisa H., Longland, William S., Murphy, Dennis D. and Sedinger, James S.	Oikos	2011
**GROWTH**	Bruna, E. M., Nardy, O., Strauss, S. Y. and Harrison, S.	Journal of Ecology	2002
	Spiesman, Brian J., Bennett, Ashley, Isaacs, Rufus and Gratton, Claudio	Biological Conservation	2017
	Cascante, A., Quesada, M., Lobo, J. J. and Fuchs, E. A.	Conservation Biology	2002
	Zartman, C. E. and Jonathan Shaw, A.	American Naturalist	2006
	Bruna, E. M.	Ecology	2003
	Ryan, M. R., Burger, L. W., Jones, D. P. and Wywialowski, A. P.	American Midland Naturalist	1998
	Hanski, I. and Saccheri, I.	PLoS Biology	2006
	Barbeta, Adria, Penuelas, Josep, Ogaya, Roma and Jump, Alistair S.	Forest Ecology and Management	2011
	Vander Haegen, W. Matthew	Ecological Applications	2007
	Gagnon, Paul R., Bruna, Emilio M., Rubim, Paulo, Darrigo, Maria Rosa, Littell, Ramon C., Uriarte, Maria and Kress, W. John	Biological Conservation	2011
	González-Di Pierro, A. M., Benítez-Malvido, J., Méndez-Toribio, M., Zermeño, I., Arroyo-Rodríguez, V., Stoner, K. E. and Estrada, A.	Biotropica	2011

Appendix S4. Included publications in review of effects from habitat features, sorted by relevant ecological process.

	Oliver, Tom H., Marshall, Harry H., Morecroft, Mike D., Brereton, Tom, Prudhomme, Christel and Huntingford, Chris	Nature Climate Change	2015
	Matesanz, Silvia, Escudero, Adrian and Valladares, Fernando	Ecology	2009
	Piessens, Katrien, Adriaens, Dries, Jacquemyn, Hans and Honnay, Olivier	Oecologia	2009
	Berggren, A., Carlson, A. and Kindvall, O.	Journal of Animal Ecology	2001
	Conlisk, Erin, Motheral, Sara, Chung, Rosa, Wisinski, Colleen and Endress, Bryan	Biological Conservation	2014
	Bowers, M. A. and Dooley, J. L.	Landscape Ecology	1999
	Bruna, E. M. and Oli, M. K.	Ecology	2005
**IMMIGRATION**	Wang, Hongfang, Sork, Victoria L., Wu, Jianguo and Ge, Jianping	Forest Ecology and Management	2010
	Cascante-Marin, Alfredo, Von Meijenfeldt, Noemi, De Leeuw, Hanneke M. H., Wolf, Jan H. D., Oostermeijer, J. Gerard B. and Den Nijs, Joannes C. M.	Journal of Tropical Ecology	2009
	Matthysen, E. and Adriaensen, F.	Auk	1998
	Jangjoo, Maryam, Matter, Stephen F., Roland, Jens and Keyghobadi, Nusha	Proceedings of the National Academy of Sciences of the United States of America	2016
	Cooper, Caren B., Walters, Jeffrey R. and Ford, Hugh	Emu	2002
	G. Rabasa, S., Gutiérrez, D. and Escudero, A.	Oikos	2007
	Holland, Greg J. and Bennett, Andrew F.	Austral Ecology	2011
	Pierre, Matthew J. St and Hendrix, Stephen D.	Prairie Naturalist	2004
	Klenner, W. and Sullivan, T. P.	Forest Ecology and Management	2009
	Matter, S. F., Roland, J., Keyghobadi, N. and Sabourin, K.	American Midland Naturalist	2003
	Holland, Greg J. and Bennett, Andrew F.	Ecography	2010
	Hoebee, S. E., Arnold, U., Dueggelin, C., Gugerli, F., Brodbeck, S., Rotach, P. and Holderegger, R.	Heredity	2007
	Kotze, D. Johan and Lawes, Michael J.	Austral Ecology	2007
	Krauss, J., Steffan-Dewenter, I. and Tscharntke, T.	Oecologia	2003
	Anderson, Christine S. and Meikle, Douglas B.	Conservation Genetics	2010
	Millar, Melissa A., Coates, David J. and Byrne, Margaret	Annals of Botany	2014

Appendix S4. Included publications in review of effects from habitat features, sorted by relevant ecological process.

	Bergman, K. O. and Landin, J.	Biological Conservation	2001
	Mora, Matias S., Mapelli, Fernando J., Gaggiotti, Oscar E., Kittlein, Marcelo J. and Lessa, Enrique P.	BMC Genetics	2010
	Bonelli, Simona, Vrabec, Vladimir, Witek, Magdalena, Barbero, Francesca, Patricelli, Dario and Nowicki, Piotr	Population Ecology	2013
	Bowers, M. A. and Dooley, J. L.	Landscape Ecology	1999
	Bueno-Enciso, Javier, Ferrer, Esperanza S., Barrientos, Rafael, Serrano-Davies, Eva and Jose Sanz, Juan	Acta Oecologica-International Journal of Ecology	2016
	Akeboshi, A., Takagi, S., Murakami, M., Hasegawa, M. and Miyashita, T.	Journal of Insect Conservation	2015
	Baguette, M., Mennechez, G., Petit, S. and Schtickzelle, N.	Comptes Rendus Biologies	2003
	Banks, Sam C. and Lindenmayer, David B.	Journal of Animal Ecology	2014
**MORTALITY**	Schmitz, R. A. and Clark, W. R.	Journal of Wildlife Management	1999
	Jonsson, Mari T., Fraver, Shawn, Jonsson, Bengt Gunnar, Dynesius, Mats, Rydgard, Mats and Esseen, Per-Anders	Forest Ecology and Management	2007
	Zartman, C. E. and Jonathan Shaw, A.	American Naturalist	2006
	Rush, S. A. and Stutchbury, B. J. M.	Auk	2008
	King, Andrew J., Melbourne, Brett A., Davies, Kendi F., Nicholls, A. O., Austin, Mike P., Tuff, Kika T., Evans, Maldwyn J., Hardy, Chris M. and Cunningham, Saul A.	Landscape Ecology	2018
	Haas, Sarah E., Cushman, J. Hall, Dillon, Whalen W., Rank, Nathan E., Rizzo, David M. and Meentemeyer, Ross K.	Ecology	2016
	Barbeta, Adria, Penuelas, Josep, Ogaya, Roma and Jump, Alistair S.	Forest Ecology and Management	2011
	G. Rabasa, S., Gutiérrez, D. and Escudero, A.	Oikos	2007
	Mceuen, Amy B. and Curran, Lisa M.	Plant Ecology	2006
	Hicks, Joseph P., Hails, Rosemary S. and Sait, Steven M.	Landscape Ecology	2015
	Currie, D. and Matthysen, E.	Belgian Journal of Zoology	1998
	Oliver, Tom H., Marshall, Harry H., Morecroft, Mike D., Brereton, Tom, Prudhomme, Christel and Huntingford, Chris	Nature Climate Change	2015
	Stangler, Eva S., Hanson, Paul E. and Steffan-Dewenter, Ingolf	Apidologie	2016

Appendix S4. Included publications in review of effects from habitat features, sorted by relevant ecological process.

	Cornelius, Cintia, Awade, Marcelo, Candia-Gallardo, Carlos, Sieving, Kathryn E. and Metzger, Jean Paul	Perspectives in Ecology and Conservation	2017
	Wilder, S. M., Abtahi, A. M. and Meikle, D. B.	American Midland Naturalist	2005
	Apps, Clayton D., Mclellan, Bruce N., Kinley, Trevor A., Serrouya, Robert, Seip, Dale R. and Wittmer, Heiko U.	Journal of Wildlife Management	2013
	Cook, W. M., Holt, R. D. and Yao, J.	Oecologia	2001
	Bonelli, Simona, Vrabec, Vladimir, Witek, Magdalena, Barbero, Francesca, Patricelli, Dario and Nowicki, Piotr	Population Ecology	2013
	Chen, Qiong, Tomlinson, Kyle W., Cao, Lin and Wang, Bo	Integrative Zoology	2017
	Becker, C. G. and Zamudio, K. R.	Proceedings of the National Academy of Sciences of the United States of America	2011
	Chavez-Pesqueira, Mariana and Nunez-Farfan, Juan	Biotropica	2016
	Benitez-Malvido, J., Garcia-Guzman, G. and Kossmann-Ferraz, I. D.	Biological Conservation	1999
**PERSISTENCE**	Sarre, S. D.	Journal of Herpetology	1998
	Turlure, Camille, Choutt, Julie, Van Dyck, Hans, Baguette, Michel and Schtickzelle, Nicolas	Journal of Insect Conservation	2010
	Henein, K., Wegner, J. and Merriam, G.	Oikos	1998
	Fernández-Juricic, E.	Landscape and Urban Planning	2004
	Day, J. R. and Possingham, H. P.	Theoretical Population Biology	1995
	Rannap, Riinu, Lohmus, Asko and Jakobson, Kaidi	Wetlands	2007
	Collins, Cathy D., Holt, Robert D. and Foster, Bryan L.	Ecology	2009
	Borgella, R. and Gavin, T. A.	Ecological Applications	2005
	Krauss, J., Steffan-Dewenter, I. and Tscharntke, T.	Oecologia	2003
	Hames, R. S., Rosenberg, K. V., Lowe, J. D. and Dhondt, A. A.	Journal of Animal Ecology	2001
	D'amore, Antonia, Hemingway, Valentine and Wasson, Kerstin	Biological Invasions	2010
	Alderman, J., Mccollin, D., Hinsley, S. A., Bellamy, P. E., Picton, P. and Crockett, R.	Landscape Ecology	2005

Appendix S4. Included publications in review of effects from habitat features, sorted by relevant ecological process.

	Beever, E. A., Brussard, P. F. and Berger, J.	Journal of Mammalogy	2003
	Beever, Erik A., Perrine, John D., Rickman, Tom, Flores, Mary, Clark, John P., Waters, Cassie, Weber, Shana S., Yardley, Braden, Thoma, David, Chesley-Preston, Tara, Goehring, Kenneth E., Magnuson, Michael, Nordensten, Nancy, Nelson, Melissa and Collins, Gail H.	Journal of Mammalogy	2016
**REPRODUCTION**	Wang, Hongfang, Sork, Victoria L., Wu, Jianguo and Ge, Jianping	Forest Ecology and Management	2010
	Bruna, E. M., Nardy, O., Strauss, S. Y. and Harrison, S.	Journal of Ecology	2002
	Cascante-Marin, Alfredo, Von Meijenfeldt, Noemi, De Leeuw, Hanneke M. H., Wolf, Jan H. D., Oostermeijer, J. Gerard B. and Den Nijs, Joannes C. M.	Journal of Tropical Ecology	2009
	Richmond, S., Nol, E. and Burke, D.	Canadian Journal of Zoology-Revue Canadienne De Zoologie	2011
	Wolf, A. T. and Harrison, S. P.	Conservation Biology	2001
	Spanhove, T., Lehouck, V., Boets, P. and Lens, L.	Animal Conservation	2009
	Turlure, Camille, Choutt, Julie, Van Dyck, Hans, Baguette, Michel and Schtickzelle, Nicolas	Journal of Insect Conservation	2010
	Scridel, Davide, Groom, Jonathan D. and Douglas, David J. T.	Bird Study	2017
	Cascante, A., Quesada, M., Lobo, J. J. and Fuchs, E. A.	Conservation Biology	2002
	Rabasa, Sonia G., Gutierrez, David and Escudero, Adrian	Plant Ecology	2009
	Mimura, Makiko, Barbour, Robert C., Potts, Brad M., Vaillancourt, Rene E. and Watanabe, Kazuo N.	Molecular Ecology	2009
	Lee, M., Fahrig, L., Freemark, K. and Currie, D. J.	Oikos	2002
	Cornell, Kerri L. and Donovan, Therese M.	Landscape Ecology	2010
	Hadley, Adam S., Frey, Sarah J. K., Robinson, W. Douglas, Kress, W. John and Betts, Matthew G.	Ecology	2014
	Weldon, Aimee J.	Conservation Biology	2006
	Rush, S. A. and Stutchbury, B. J. M.	Auk	2008
	Matthysen, E. and Adriaensen, F.	Auk	1998
	Zharikov, Yuri, Lank, David B. and Cooke, Fred	Journal of Applied Ecology	2007
	Valdivia, C. E., Simonetti, J. A. and Henriquez, C. A.	Biodiversity and Conservation	2006
	Cooper, Caren B., Walters, Jeffrey R. and Ford, Hugh	Emu	2002

Appendix S4. Included publications in review of effects from habitat features, sorted by relevant ecological process.

Winter, M., Johnson, D. H., Shaffer, J. A., Donovan, T. M. and Svedarsky, W. D.	Journal of Wildlife Management	2006
Sosa, R. A. and Lopez De Casenave, J.	Ecological Research	2017
O'connell, Lisa M., Mosseler, Alex and Rajora, Om P.	Canadian Journal of Botany-Revue Canadienne De Botanique	2006
Manikowska-Slepowronska, Brygida, Slepowronski, Krzysztof and Jakubas, Dariusz	Polish Journal of Ecology	2016
Carrascal, L. M., Bautista, L. M. and Lazaro, E.	Biological Conservation	1993
Ryan, M. R., Burger, L. W., Jones, D. P. and Wywialowski, A. P.	American Midland Naturalist	1998
Carlson, A. and Hartman, G.	Biodiversity and Conservation	2001
Magrach, Ainhoa, Larrinaga, Asier R. and Santamaria, Luis	Conservation Biology	2012
Gardiner, Mary M., O'neal, Matthew E. and Landis, Douglas A.	Plos One	2011
Barbeta, Adria, Penuelas, Josep, Ogaya, Roma and Jump, Alistair S.	Forest Ecology and Management	2011
Huhta, E., Jokimaki, J. and Helle, P.	Ecography	1998
Meyer, Joseph S., Irwin, Larry L. and Boyce, Mark S.	Wildlife Monographs	1998
Bruna, E. M.	Oecologia	2002
Oster, Mathias and Eriksson, Ove	Ecoscience	2007
Le Tortorec, Eric, Helle, Samuli, Kayhko, Niina, Suorsa, Petri, Huhta, Esa and Hakkarainen, Harri	Journal of Animal Ecology	2013
Soga, Masashi and Koike, Shinsuke	Plos One	2013
Ghazoul, J. and Mcleish, M.	Plant Ecology	2001
Langen, T. A., Bolger, D. T. and Case, T. J.	Oecologia	1991
Hermansen, T. D., Ayre, D. J. and Minchinton, T. E.	Plant Ecology	2014
Donovan, T. M., Thompson, F. R., Faaborg, J. and Probst, J. R.	Conservation Biology	1995
Vander Haegen, W. Matthew	Ecological Applications	2007
Nunez-Avila, Mariela C., Uriarte, Maria, Marquet, Pablo A. and Armesto, Juan J.	Journal of Ecology	2013
Wong, T. C. M., Sodhi, N. S. and Turner, I. M.	Biological Conservation	1998
Naoe, Shoji, Sakai, Shoko, Sawa, Ayako and Masaki, Takashi	Ecological Research	2011
Storch, I.	Ornis Scandinavica	1991

Appendix S4. Included publications in review of effects from habitat features, sorted by relevant ecological process.

Gagnon, Paul R., Bruna, Emilio M., Rubim, Paulo, Darrigo, Maria Rosa, Littell, Ramon C., Uriarte, Maria and Kress, W. John	Biological Conservation	2011
Klenner, W. and Sullivan, T. P.	Forest Ecology and Management	2009
Robles, Huco, Ciudad, Carlos, Vera, Ruben, Olea, Pedro P. and Matthysen, Erik	Auk	2008
Vanhorn, M. A., Gentry, R. M. and Faaborg, J.	Auk	1995
King, D. I. and Degraaf, R. M.	Forest Ecology and Management	2004
Duncan, D. H., Nicotra, A. B., Wood, J. T. and Cunningham, S. A.	Journal of Ecology	2004
Ison, Jennifer L. and Wagenius, Stuart	Journal of Ecology	2014
Santos, T., Telleria, J. L. and Virgos, E.	Ecography	1999
Falk, Karla J., Nol, Erica and Burke, Dawn M.	Landscape Ecology	2011
Wolf, A.	Biological Conservation	2001
Lienert, J. and Fischer, M.	Basic and Applied Ecology	2004
Schmucki, Reto and De Blois, Sylvie	Oecologia	2009
Ashworth, Lorena, Calvino, Ana, Leticia Marti, Maria and Aguilar, Ramiro	Austral Ecology	2015
Serio-Silva, J. C. and Rico-Gray, V.	Oryx	2002
Mceuen, Amy B. and Curran, Lisa M.	Plant Ecology	2006
Winiarski, Jason M., Moorman, Christopher E., Carpenter, John P. and Hess, George R.	Ecosphere	2017
Wilder, S. M. and Meikle, D. B.	Journal of Mammalogy	2006
Hicks, Joseph P., Hails, Rosemary S. and Sait, Steven M.	Landscape Ecology	2015
Orrock, J. L. and Damschen, E. I.	Ecological Applications	2005
Arangovelez, N. and Kattan, G. H.	Biological Conservation	1997
Krauss, Siegfried L., Hermanutz, Luise, Hopper, Stephen D. and Coates, David J.	Australian Journal of Botany	2007
González-Di Pierro, A. M., Benítez-Malvido, J., Méndez-Toribio, M., Zermeño, I., Arroyo-Rodríguez, V., Stoner, K. E. and Estrada, A.	Biotropica	2011
Seltmann, Peggy, Cocucci, Andrea, Renison, Daniel, Cierjacks, Arne and Hensen, Isabell	Basic and Applied Ecology	2009
Githiru, M., Lens, L. and Cresswell, W.	Biological Conservation	2005
Vesk, Peter A., Davidson, Anthony and Chee, Yung En	Austral Ecology	2010
Garvey, Mary E., Nol, Erica, Howerter, David W. and Armstrong, Llwellyn M.	Condor	2013

Appendix S4. Included publications in review of effects from habitat features, sorted by relevant ecological process.

Umapathy, G. and Kumar, A.	Primates	2000
Matthysen, E.	Oecologia	1999
Matesanz, Silvia, Escudero, Adrian and Valladares, Fernando	Ecology	2009
Lens, L. and Dhondt, A. A.	Ibis	1994
Garcia, Daniel, Martinez, Daniel, Herrera, Jose M. and Morales, Juan M.	Ecography	2013
Hoebee, S. E., Arnold, U., Dueggelin, C., Gugerli, F., Brodbeck, S., Rotach, P. and Holderegger, R.	Heredity	2007
Pauw, Anton	Ecology	2007
Cox, W. Andrew, Thompson, Frank R., Iii, Reidy, Jennifer L. and Faaborg, John	Global Change Biology	2013
Stangler, Eva S., Hanson, Paul E. and Steffan-Dewenter, Ingolf	Apidologie	2016
Taki, Hisatomo, Kevan, Peter G. and Yamaura, Yuichi	Canadian Field-Naturalist	2008
Kotze, D. Johan and Lawes, Michael J.	Austral Ecology	2007
Sugiyama, Anna and Peterson, Chris J.	Plant Ecology	2013
Seifert, Birgit and Fischer, Markus	Biological Conservation	2010
Kolb, Annette	Biological Conservation	2008
Groom, M. J. and Preuninger, T. E.	Evolutionary Ecology	2000
Lloyd, P., Martin, T. E., Redmond, R. L., Langner, U. and Hart, M. M.	Ecological Applications	2005
Kikuchi, Satoshi, Shibata, Mitsue and Tanaka, Hiroshi	Global Ecology and Conservation	2015
Peak, Rebecca G.	Condor	2007
Hersek, M. J., Frankel, M. A., Cigliano, J. A. and Wasserman, F. E.	Auk	2002
Huhta, E., Aho, T., Jäntti, A., Suorsa, P., Kuitunen, M., Nikula, A. and Hakkarainen, H.	Conservation Biology	2004
Thiele, Jan, Schuckert, Ulrike and Otte, Annette	Landscape Ecology	2008
Gonzalez-Varo, Juan P., Arroyo, Juan and Aparicio, Abelardo	Biological Conservation	2009
Garcia, Daniel and Chacoff, Natacha P.	Conservation Biology	2007
Dunley, Bernardo S., Freitas, Leandro and Galetto, Leonardo	Biotropica	2009
Fuchs, E. J., Lobo, J. A. and Quesada, M.	Conservation Biology	2003
Santos, T. and Telleria, J. L.	Forest Ecology and Management	1997
Castilleja Sanchez, Paty, Delgado Valerio, Patricia, Saenz-Romero, Cuauhtemoc and Herrerias Diego, Yvonne	Forests	2016
Aizen, M. A. and Feinsinger, P.	Ecology	1994

Appendix S4. Included publications in review of effects from habitat features, sorted by relevant ecological process.

Newmark, William D. and Stanley, Thomas R.	Proceedings of the National Academy of Sciences of the United States of America	2011
Brudvig, Lars A., Damschen, Ellen I., Haddad, Nick M., Levey, Douglas J. and Tewksbury, Joshua J.	Ecology	2015
Braun, Marcus and Gottsberger, Gerhard	Nordic Journal of Botany	2012
D'amore, Antonia, Hemingway, Valentine and Wasson, Kerstin	Biological Invasions	2010
Chapman, C. A., Chapman, L. J., Vulinec, K., Zanne, A. and Lawes, M. J.	Biotropica	2003
Kossenko, Serguei M. and Kaygorodova, Evgeniya Yu	Ardea	2007
Piessens, Katrien, Adriaens, Dries, Jacquemyn, Hans and Honnay, Olivier	Oecologia	2009
Marsh, D. M.	Ecology	2001
Bennett, Joanne M., Clarke, Rohan H., Thomson, James R. and Mac Nally, Ralph	Ecography	2015
Cottam, Michael R., Robinson, Scott K., Heske, Edward J., Brawn, Jeffrey D. and Rowe, Kevin C.	Biological Conservation	2009
Mazgajski, Tomasz D. and Rejt, Lukasz	Annales Zoologici Fennici	2006
Skogen, Krissa A., Jogesh, Tania, Hilpman, Evan T., Todd, Sadie L., Rhodes, Matthew K., Still, Shannon M. and Fant, Jeremie B.	American Journal of Botany	2016
Hanson, Thor, Brunsfeld, Steven, Finegan, Bryan and Waits, Lisette	Journal of Tropical Ecology	2007
Yates, Colin J., Coates, David J., Elliott, Carole and Byrne, Margaret	Biodiversity and Conservation	2007
Bergin, T. M., Best, L. B., Freemark, K. E. and Koehler, K. J.	Landscape Ecology	2000
Aguilar, Ramiro, Ashworth, Lorena, Calvino, Ana and Quesada, Mauricio	Biological Conservation	2012
Coudrain, Valerie, Herzog, Felix and Entling, Martin H.	Plos One	2013
Burke, D. M. and Nol, E.	Ecological Applications	2000
Bernath-Plaisted, Jacy, Nenninger, Heather and Koper, Nicola	Royal Society Open Science	2017
Cunningham, S. A.	Conservation Biology	2000
Cranmer, Louise, Mccollin, Duncan and Ollerton, Jeff	Oikos	2012
Aguirre, Armando and Dirzo, Rodolfo	Biological Conservation	2008
Askins, Robert A., Zuckerberg, Benjamin and Novak, Leah	Forest Ecology and Management	2007
Cardozo, G. and Chiaraviglio, M.	Biological Conservation	2008

Appendix S4. Included publications in review of effects from habitat features, sorted by relevant ecological process.

Ball, I. J., Eng, R. L. and Ball, S. K.	Wildlife Society Bulletin	1995
Bayne, E. M. and Hobson, K. A.	Auk	2001
Chapa-Vargas, Leonardo and Robinson, Scott K.	Auk	2007
Bain, Glen C., Hall, Michelle L. and Mulder, Raoul A.	Molecular Ecology	2014
Cayuela, Hugo, Cheylan, Marc and Joly, Pierre	Amphibia-Reptilia	2011
Blaauw, Brett R. and Isaacs, Rufus	Basic and Applied Ecology	2014
Bowers, M. A., Gregario, K., Brame, C. J., Matter, S. F. and Dooley, J. L.	Oecologia	1996
Brown, Jessi L., Collopy, Michael W. and Smallwood, John A.	Bird Conservation International	2014
Bueno-Enciso, Javier, Ferrer, Esperanza S., Barrientos, Rafael, Serrano-Davies, Eva and Jose Sanz, Juan	Acta Oecologica-International Journal of Ecology	2016
Boulton, Rebecca L., Richard, Yvan and Armstrong, Doug P.	Biological Conservation	2008
Barber, J. D., Wiggers, E. P. and Renken, R. B.	Journal of Wildlife Management	1998
Chavez-Pesqueira, Mariana and Nunez-Farfan, Juan	Biotropica	2016
Alberto Puig-Lagunes, Angel, Canales-Espinosa, Domingo, Rangel-Negrin, Ariadna and Dias, Pedro Americo D.	International Journal of Primatology	2016
Bruna, E. M. and Oli, M. K.	Ecology	2005
Aquilani, S. M. and Brewer, J. S.	Natural Areas Journal	2004
Cramer, J. M., Mesquita, R. C. G., Bentos, T. V., Moser, B. and Williamson, G. B.	Biotropica	2007
Bonifait, Sylvain and Villard, Marc-Andre	Ecography	2010
Adriaens, Dries, Jacquemyn, Hans, Honnay, Olivier and Hermy, Martin	Acta Oecologica-International Journal of Ecology	2009
Cordeiro, Norbert J., Ndangalasi, Henry J., Mcentee, Jay P. and Howe, Henry F.	Ecology	2009
Aguilar, R. and Galetto, L.	Oecologia	2004
Ashworth, Lorena and Marti, Maria L.	Biotropica	2011
Benitez-Malvido, J.	Conservation Biology	1998
Chapa-Vargas, Leonardo and Robinson, Scott K.	Landscape Ecology	2006
Braun, Marcus, Piechowski, Daniel, Kazda, Marian and Gottsberger, Gerhard	Journal of Tropical Ecology	2012

Appendix S4. Included publications in review of effects from habitat features, sorted by relevant ecological process.

	Bayne, E. M. and Hobson, K. A.	Ecology	2002
	Bruna, E. M. and Kress, W. J.	Conservation Biology	2002
	Coudrain, Valerie, Rittiner, Sarah, Herzog, Felix, Tinner, Willy and Entling, Martin H.	Insect Science	2016
	Andreazzi, C. S., Pimenta, C. S., Pires, A. S., Fernandez, F. A. S., Oliveira-Santos, L. G. and Menezes, J. F. S.	Biotropica	2012
	Alberto Sosa, Ramon and Lopez De Casenave, Javier	Ecological Research	2017
	Conenna, Irene, Valkama, Jari and Chamberlain, Dan	Journal of Ornithology	2017
	Armstrong, D. P., Mcarthur, N., Govella, S., Morgan, K., Johnston, R., Gorman, N., Pike, R. and Richard, Y.	Biological Conservation	2013
	Cornelius, Cintia	Condor	2008
	Chaves, Oscar M., Stoner, Kathryn E., Arroyo-Rodriguez, Victor and Estrada, Alejandro	International Journal of Primatology	2011
	Balent, K. L. and Norment, C. J.	Journal of Field Ornithology	2003
	Chen, Min and Zhao, Xue-Yong	Ecology and Evolution	2017
	Burrough, Reuben, Conroy, Gabriel, Lamont, Robert W., Shimizu-Kimura, Yoko and Shapcott, Alison	Australian Journal of Botany	2018
	Burger, L. D., Burger, L. W. and Faaborg, J.	Journal of Wildlife Management	1994
	Barrios, Beyte, Arellano, Gabriel and Koptur, Suzanne	Plant Ecology	2011
	Burgos, Aracely, Grez, Audrey A. and Bustamante, Ramiro O.	Forest Ecology and Management	2008
**SURVIVAL**	Grovenburg, Troy W., Klaver, Robert W. and Jenks, Jonathan A.	Journal of Wildlife Management	2012
	Robles, Hugo, Ciudad, Carlos, Vera, Ruben and Baglione, Vittorio	Ecography	2007
	Henein, K., Wegner, J. and Merriam, G.	Oikos	1998
	Nupp, T. E. and Swihart, R. K.	Journal of Mammalogy	1998
	Cooper, Caren B., Walters, Jeffrey R. and Ford, Hugh	Emu	2002
	Bruna, E. M.	Ecology	2003
	Lawes, Timothy J., Anthony, Robert G., Robinson, W. Douglas, Forbes, James T. and Lorton, Glenn A.	Western North American Naturalist	2012
	Klenner, W. and Sullivan, T. P.	Forest Ecology and Management	2009
	Johannesen, E. and Ims, R. A.	Ecology	1996

Appendix S4. Included publications in review of effects from habitat features, sorted by relevant ecological process.

	Vandewoestijne, Sofie, Schtickzelle, Nicolas and Baguette, Michel	Bmc Biology	2008
	Holland, Greg J. and Bennett, Andrew F.	Ecography	2010
	Matthysen, E.	Oecologia	1999
	Matesanz, Silvia, Escudero, Adrian and Valladares, Fernando	Ecology	2009
	Peter, Franziska, Berens, Dana G., Grieve, Graham R. and Farwig, Nina	Biotropica	2015
	Kobayashi, Takato, Nakashizuka, Tohru and Kitahara, Masahiko	Ecological Research	2009
	Bayne, E. M. and Hobson, K. A.	Ecology	2002
	Baguette, M., Mennechez, G., Petit, S. and Schtickzelle, N.	Comptes Rendus Biologies	2003
	Andreazzi, C. S., Pimenta, C. S., Pires, A. S., Fernandez, F. A. S., Oliveira-Santos, L. G. and Menezes, J. F. S.	Biotropica	2012
	Amos, J. N., Balasubramaniam, S., Grootendorst, L., Harrisson, K. A., Lill, A., Mac Nally, R., Pavlova, A., Radford, J. Q., Takeuchi, N., Thomson, J. R. and Sunnucks, P.	Journal of Avian Biology	2013
